# The Biological Characteristics and Mouse Model of Lassa Virus From the First Imported Case in China

**DOI:** 10.1002/mco2.70315

**Published:** 2025-08-03

**Authors:** Yanan Zhou, Junbin Wang, Ranran Cao, Yun Yang, Yuliang Feng, Cong Tang, Hao Yang, Qing Huang, Wenhai Yu, Haixuan Wang, Jiandong Shi, Kaiyun Ding, Longhai Yuan, Qing Dai, Xingping Zhao, Haiyan Li, Mengli Yang, Fangyu Luo, Fanli Zhu, Yong Zhang, Daoju Wu, Xiaorong Yang, Shuaiyao Lu, Qiangming Sun, Li Zhang, Youchun Wang

**Affiliations:** ^1^ National Kunming High‐Level Biosafety Primate Research Center，State Key Laboratory of Respiratory Health and Multimorbidity Institute of Medical Biology Chinese Academy of Medical Sciences and Peking Union Medical College Kunming China; ^2^ Sichuan Center for Disease Control and Prevention Chengdu China; ^3^ Key Laboratory of Pathogen Infection Prevention and Control (Peking Union Medical College) Ministry of Education Beijing China; ^4^ Yunnan Key Laboratory of Cross‐Border Infectious Disease Control and Prevention and Novel Drug Development Kunming China

**Keywords:** biological characteristics, Lassa virus, mouse infection model, virus isolation

## Abstract

Lassa fever (LF) is a fatal hemorrhagic disease caused by the Lassa virus (LASV), which mainly spreads in Africa. As China's interactions with Africa become more frequent, the risk of LF being imported into China also rises, making the study of LASV increasingly urgent. In this study, the Lineage IV LASV strain was successfully isolated from the first imported case in China. Compared with the LASV genome, the isolated strain may exhibit greater infectivity and interspecies transmission capabilities. We successfully established BALB/c, C57BL/6, and AG129 mouse infection models and found that intranasal inoculation was the most stable infection method. Select the anti‐LASV drug LHF‐535 for preliminary evaluation, further confirming the stability of the model. In summary, the isolated strain exhibits enhanced transmission capabilities and may spread between mice via the respiratory tract, meriting greater attention and emphasis. This study will bridge the gap in China's independent P4‐level pathogen isolation, meet national biosafety and strategic needs, and provide certain support for LASV research.

## Graphical abstract

This is the first case of Lassa fever being introduced to China. A strain of the Lineage IV LASV was successfully isolated, and genomic and mutation analysis were conducted. Infection models were constructed using BALB/c, C57BL/6, and AG129 mice, which were successfully established. LHF‐535 was utilized to further confirm the stability of the mouse model.

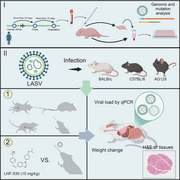

## 1 Introduction

Lassa fever (LF) is a fatal hemorrhagic disease caused by the Lassa virus (LASV), transmitted by the “multimammate rat,” also known as *Mastomys natalensis* (*Mastomys*). It is prevalent across sub‐Saharan Africa [1]. Other rodent species, such as *Hylomyscus pamfi* and *Mastomys erythroleucus*, can also contract LASV [2]. The virus causes zoonotic infections in humans through various means, including the consumption of contaminated food and/or water, direct contact with rodent waste, inhalation of aerosols containing rodent waste, rodent bites, and direct interpersonal transmission via contact with bodily fluids [3, 4]. It is estimated that approximately 500,000 infections occur in West Africa annually, with around 10,000 deaths each year [5]. The case fatality rate for hospitalized cases can reach as high as 15%–20%, posing a significant public health threat to the affected regions [6, 7]. Nevertheless, these figures might be conservative because of the lack of standardized surveillance systems for LASV, the use of relatively rudimentary estimation methods, and the potential for misdiagnosis. Research indicates that the annual number of LF cases could be as high as 900,000 [8]. Although LASV is mainly concentrated in West Africa, it can be exported to countries outside Africa through human‐to‐human transmission, posing a potential global risk [6, 9, 10].

Nigeria experienced a large‐scale outbreak of LF in 2023, with 4702 suspected cases, 5 probable cases, and 877 confirmed cases between Epidemiological Weeks 1 and 15. Among the confirmed cases, there were 152 deaths, yielding a case fatality rate of 17%. In comparison to the number of confirmed cases reported during the same period in 2022 (733 cases), the number increased by 20% [11]. For the entire year of 2024 in Nigeria, there were 10,098 suspected cases, 23 probable cases, and 1309 confirmed cases. Among these confirmed cases, 214 deaths occurred, with a case fatality rate of 16.3% [12]. As China's interactions with Africa become more frequent, the risk of LF being imported into China also rises, making the study of imported cases of LASV increasingly urgent.

The LASV belongs to the *Arenaviridae* family and is classified as a negative‐strand ribonucleic acid (RNA) virus. Its genome comprises two segments: the large (L) and the small (S) RNA segments. The L segment encodes the RNA‐dependent RNA polymerase and the Z protein gene, whereas the S segment encodes the glycoprotein (GP) and nucleoprotein (NP) genes [13]. LASV isolates display high genetic diversity at the genomic sequence level and can currently be categorized into up to seven distinct lineages [14]. LASV lineages I, II, and III are predominantly found in Nigeria, while LASV lineage IVis primarily distributed in Sierra Leone, Guinea, Liberia, and other regions. Lineages V, VI, and VII are more recently identified lineages with limited distribution. Lineage V is primarily distributed in Mali, lineage VI is mainly found in Ghana, and lineage VII is primarily distributed in Benin [8].

Despite the initial description of LASV in 1969 and over five decades having passed, a significant number of people remain at risk of infection. To date, there is still no clinically approved treatment for LF or a vaccine to prevent LASV infection. The only antiviral drug available is ribavirin, yet its therapeutic efficacy is limited [15, 16]. Favipiravir, an RdRp inhibitor, has demonstrated antiviral activity against numerous RNA viruses. Experiments in macaques have shown that favipiravir has a higher therapeutic index compared to ribavirin [17]. Moreover, the combination of favipiravir and ribavirin has shown a synergistic effect in vitro and in vivo, extending the survival time of LASV‐infected mice [18]. LHF‐535 is a small molecule viral entry inhibitor that targets the arenavirus envelope GP and has shown effective antiviral activity against various hemorrhagic fever arenaviruses, proving its efficacy in mouse and guinea pig disease models [19, 20, 21]. LF is emerging as an increasingly serious public health concern, urgently necessitating the development of new drugs or vaccines for treatment and control. However, China continues to experience a gap in the isolation of P4‐level pathogens. As a result, we conducted virus isolation on clinical samples from China's first imported LF case and successfully isolated the virus, which is urgently needed in China today.

Animal models of LASV infection are essential tools for evaluating potential antiviral compounds and treatment modalities. Common animal models include mice, guinea pigs, ferrets, and nonhuman primates (NHPs). Currently, inbred Strain 13 and outbred Hartley guinea pigs can be infected with wild‐type LASV via aerosol, subcutaneous, or intraperitoneal (i.p.) routes and are considered the preferred small animal models for LASV infection [22]. Their mortality rates are 100% and 30%, respectively [23]. Several immunodeficient mouse strains are also susceptible to LASV infection, including interferon receptor knockout (IFNAR^−/−^), chimeric IFNA^−/−^B6, human/mouse chimeric HLA‐A2.1 (humanized HHD), CBA, and STAT‐deficient (STAT1−/− mice) [22, 23]. Immunodeficient mice have been used to evaluate antiviral therapeutic drugs and vaccines [22]; however, there are certain drawbacks to their research on the pathogenesis and immunology of LASV. The cynomolgus monkey is the most commonly used NHP model and exhibits disease manifestations that closely resemble those of severe LF in humans. However, primate resources are precious and not suitable for widespread application in animal experiments. Consequently, the establishment of a laboratory wild‐type mice infection model is currently lacking. There are few reports on whether LASV infects laboratory wild‐type mice. A 1985 report [24] indicated that BALB/c mice were not susceptible to LASV through intracerebral inoculation, with a mortality rate of about 30%–60% for C57BL/6 and AKR mice. However, with LASV mutations, there are limited reports on whether new emerging LASV strains have become susceptible to laboratory wild‐type mice. Thus, we conducted a preliminary analysis of the biological characteristics and established a laboratory wild‐type mouse model for the first LASV imported into China, providing basic data for related research.

## 2 Results

### 2.1 Identification and Isolation of LASV

**FIGURE 1:**
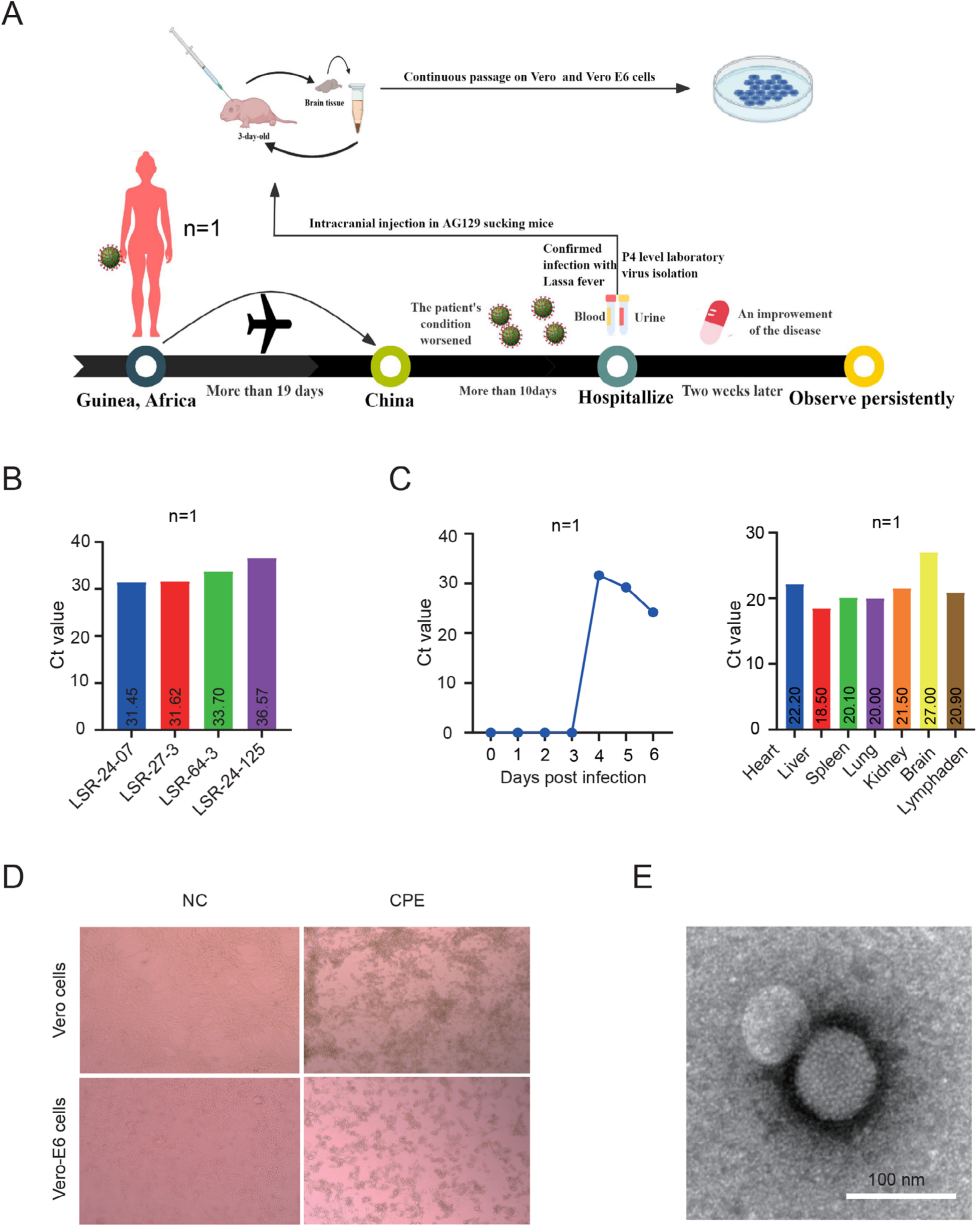
Isolation and Identification of LASV. (A) LF case situation and isolation process of LASV; (B) Ct values of LASV case samples (*n* = 1); (C) Ct values of blood and tissue samples from AG129 mice (*n* = 1) infected with LASV after isolation; (D) Cytopathic effects (CPE) of LASV infection in Vero and Vero E6 cells. Images were captured using a Leica DMiL microscope (10× objective); (E) Transmission electron microscopy observation of Lassa virus particles. Ct values = 0 indicate that it is below the detection limit, and the viral nucleic acid was not detected.

In July 2024, a case of LF was imported from Guinea, Africa, to Sichuan Province. The Sichuan Center for Disease Control and Prevention, along with other units, implemented relevant emergency measures. Following treatment, the patient's condition stabilized. Throughout the treatment period, the Sichuan Center for Disease Control and Prevention and other units collected whole blood and urine samples from the patient. The specific information of these samples is detailed in Table S1, with the cycle threshold (Ct) values of the four samples being 31.45 (whole blood), 31.62 (urine), 33.7 (urine), and 36.57 (urine) (Figure 1B). Our unit received these samples on October 14, 2024, and initiated the isolation and identification of the virus. Based on literature reports [25], our experience with virus isolation in a Bio‐Safety Level 4 laboratory (BSL‐4). We first inoculated 3‐day‐old AG129 suckling mice intracerebrally and then continuously passaged the virus. Five days postinoculation, we harvested the brains of the suckling mice, homogenized them in phosphate buffer saline (PBS), centrifuged the mixture, and subsequently utilized the supernatant for further passaging and real‐time fluorescence quantitative polymerase chain reaction (RT‐qPCR) to detect viral load. Samples that were positive for the virus were passaged in LASV‐sensitive cells (Vero and Vero E6) (Figure 1A). We successfully isolated the virus from the LSR‐24‐07 sample, and significant cytopathogenic effects (CPE) were observed in both Vero and Vero E6 cells (Figure 1D). To further confirm the successful isolation of the virus, we collected the supernatant following the cytopathic effect and inoculated it intravenously via the tail vein into AG129 mice, which are sensitive to LF [23]. We collected blood daily postinoculation to monitor for viremia and detected viremia on the fourth day. Subsequently, the Ct value began to decrease, reaching 24.2 by the sixth day. After dissection, we collected the heart, liver, spleen, lung, kidney, brain, and lymph nodes to detect viral load, and viral load was detected in all the tissues collected, with the lowest Ct value of 18.5 in the liver (Figure 1C). The viral solution was inactivated using formaldehyde, and transmission electron microscopy was employed to observe the viral particles. Particles with a diameter of approximately 100 nm were detected, similar to LASV in size and morphology [26] (Figure 1E).

### 2.2 Molecular Biological Characteristics and Receptor Affinity Prediction of LASV

**FIGURE 2:**
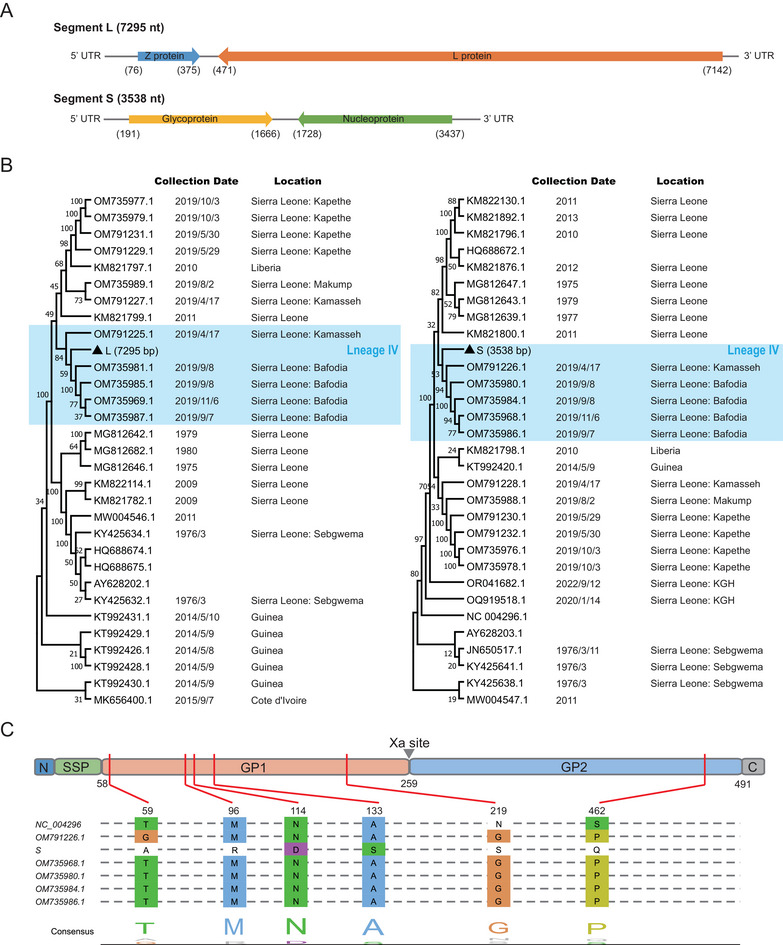
The LASV strain is shown to be lineage IV, and changes in B cell epitopes in the viral GPC protein. (A) Genome structure of the isolated strain, (B) A bootstrap phylogenetic tree of the isolated strain; Isolated‐L (left), Isolated‐S (right); (C) Amino acid variation of the glycoprotein of the isolated strain.

Two complete genome fragments were obtained and named Isolated‐L and Isolated‐S, respectively. Isolated‐L and Isolated‐S were annotated using reference genomes from the NCBI database (L: NC 004297.1, S: NC 004296.1). The Isolated‐L fragment is 7295 bp in length and contains two open reading frames (ORFs), ORF1 (76‐375) encoding the Z protein and ORF2 (471‐7142) encoding the L protein. The Isolated‐S fragment is 3538 bp in length and contains two ORFs, ORF1 (191‐1666) encoding a GP and ORF2 (1728‐3437) encoding an NP (Figure 2A). Phylogenetic trees were constructed using the top 30 close sequences for Isolated‐L and Isolated‐S. The results indicated that Isolated‐L and Isolated‐S belong to lineage IV [27]. The virus is genetically closest to the five strains circulating in Sierra Leone in 2019, which clustered within the same small branch (Figure 2B). Amino acid variation was analyzed by aligning the glycoprotein precursor (GPC) amino acid sequence of the isolated strain with the sequences of the five closest viruses and the reference genome (Figures 2C, S1). The results indicated that the isolated strain had evolved unique amino acid variations, including T59A, M96R, N114D, A133S, and N209S in GP1 and S462Q in the GP2 region (Figure 2C). Furthermore, by comparing the GPC amino acid sequence of the original patient samples, it was found that these mutation sites are consistent. Alterations may modify the B‐cell epitope of the isolated strain and affect the virus's entry into the host cell [13, 28]. L protein, NP, and Z protein amino acid sequences of the isolated strain with the sequences of the closest viruses and the reference genome (Figures S2 and S3).

**FIGURE 3:**
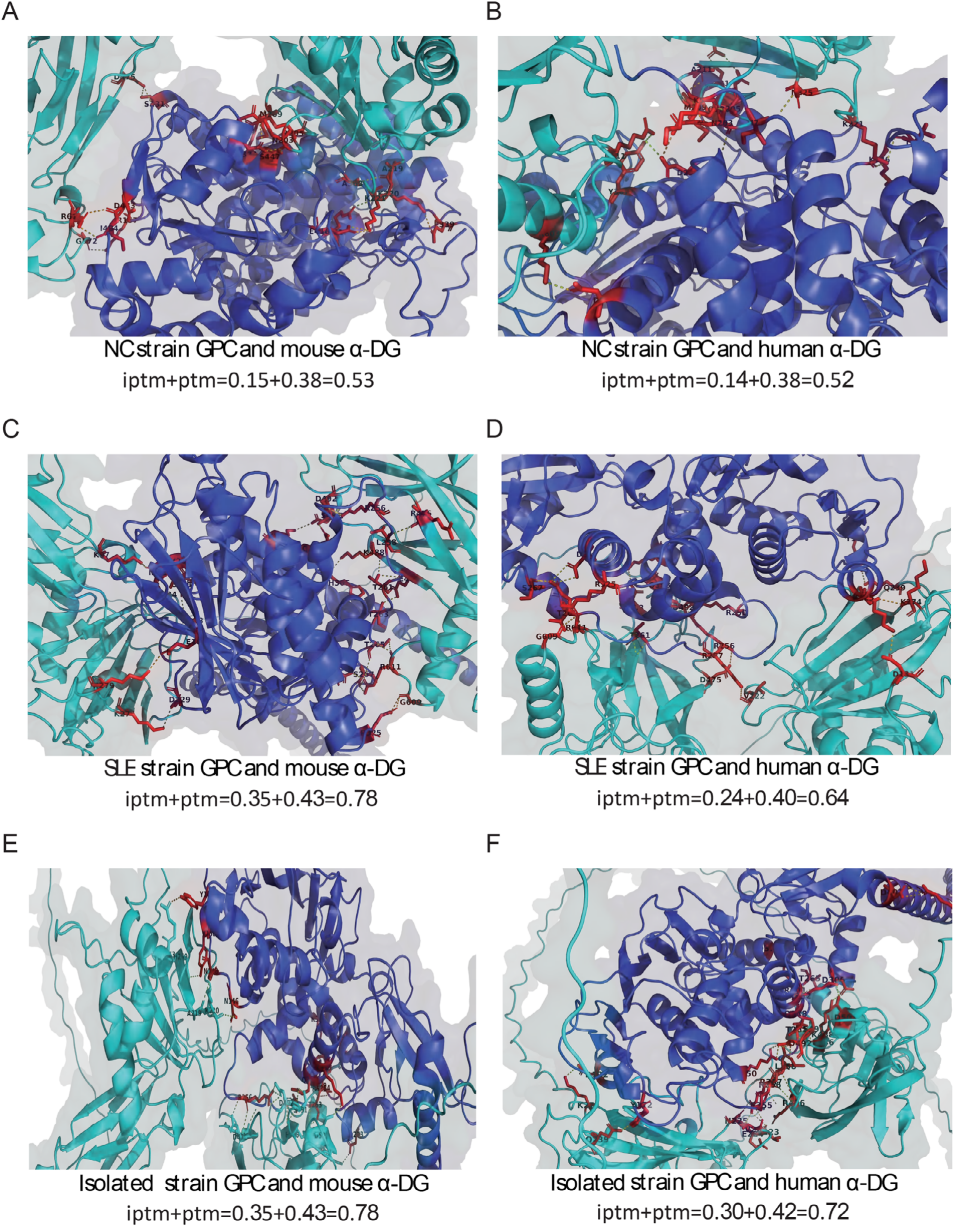
Structural simulation of the binding between different LASV strains and receptors. (A) Structural simulation of the binding between the NC strain GPC and mouse α‐DG; (B) Structural simulation of the binding between the NC strain GPC and human α‐DG; (C) Structural simulation of the binding between the SLE strain GPC and mouse α‐DG; (D) Structural simulation of the binding between the SLE strain GPC and human α‐DG; (E) Structural simulation of the binding between the isolated strain GPC and mouse α‐DG; (F) Structural simulation of the binding between the isolated strain GPC and human α‐DG. The color scheme represents deep blue for GPC, sky blue for α‐DG, and red for interaction sites. Iptm indicates interaction score (higher scores denote stronger interactions), ptm represents structural prediction accuracy (higher accuracy indicates more precise predictions), and the sum of these two values reflects the strength of the final receptor‐ligand binding ability.

The LASV GPC sequences from various strains utilized in this receptor binding analysis were sourced from NCBI, including LASV‐NC (NC_004296.1) and LASV‐SLE (Kamasseh‐08476 OM791226.1). The amino acid sequences of the LASV receptor α‐dystroglycan (α‐DG) were sourced from the UniProt database, including those from mouse (Q62165), human (Q14118), macaque (F6RU27), and guinea pig (A0A286XNA0). To preliminarily explore the differences in receptor affinity between the isolated strain and previously emerged strains, we conducted preliminary predictions of the binding ability of the GPC protein of LASV‐NC (NC), LASV‐SLE (SLE), and LASV‐Isolated to their receptors [29]. The results indicated that there was no difference in binding ability between the NC strain and the isolated strain with α‐DG receptors across various species. Moreover, the receptor binding ability of the NC strain was lower than that of the isolated strain in all species tested. The predicted receptor binding ability values for the NC strain in mouse, human, macaque, and guinea pig species are 0.53, 0.52, 0.53, and 0.53, respectively; whereas for the isolated strain, the values are 0.78, 0.72, 0.72, and 0.74, respectively (Figure 3A,B,E,F; Figure S4A,B,E,F). The predicted receptor binding capabilities of the SLE strain for mouse, human, macaque, and guinea pig species are 0.78, 0.64, 0.71, and 0.55, respectively. The SLE strain exhibited the higher binding affinity for α‐DG in both mice and rhesus monkeys, surpassing that in guinea pigs and slightly exceeding that in humans (Figure 3C,D; Figure S4C,D). Conversely, the isolated strain exhibited a greater binding affinity for the guinea pig receptor and a higher affinity for the human receptor than the SLE strain. The predicted binding ability of the isolated strain and SLE strain to human receptors is 0.72 and 0.64, respectively, while the predicted binding ability to guinea pig receptors is 0.74 and 0.55, respectively (Figure 3C,E; Figure S4D,F). This indicates that the isolated strain may possess a higher infectivity and interspecies transmission potential than the NC and SLE strains.

### 2.3 Intravenous Tail Injection Method for Establishing a LASV‐Infected Mouse Model

**FIGURE 4:**
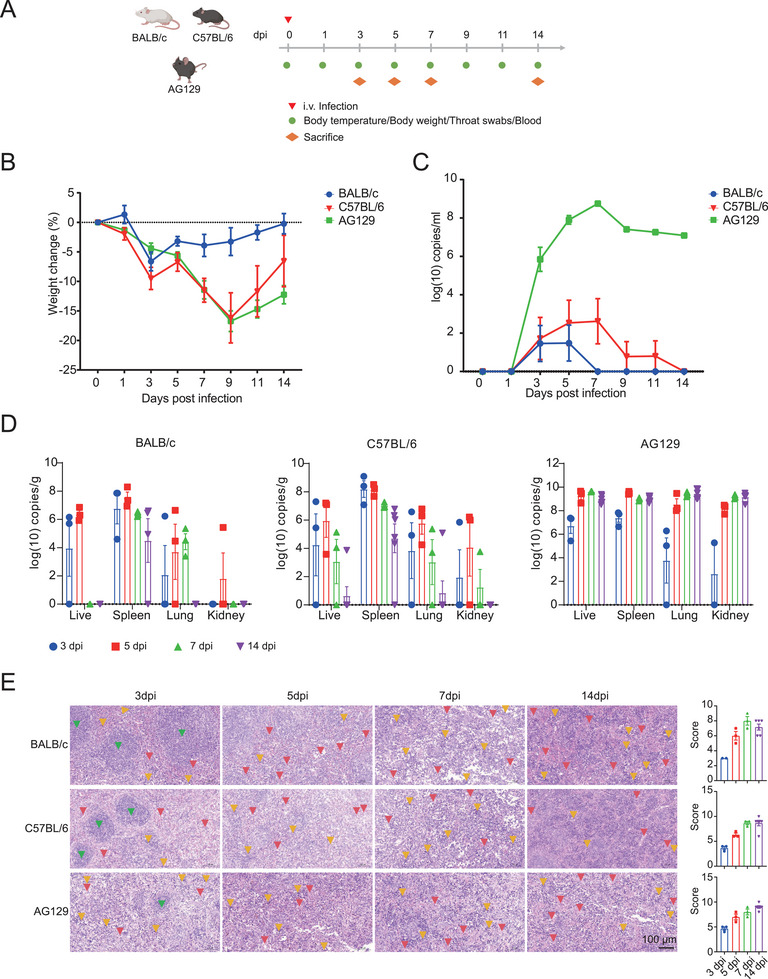
Intravenous tail infection method for infecting BALB/c, C57BL/6, and AG129 mice with LASV. (A) Schematic design of the intravenous tail infection of BALB/c, C57BL/6, and AG129 mice. Body temperature, weight, blood, and throat swabs were collected every other day postinfection. Mice were dissected on days 3, 5, 7, and 14 postinfection. (B) Changes in body weight of BALB/c, C57BL/6, and AG129 mice (*n* = 6) postinfection. (C) Changes in viral load in the blood of BALB/c, C57BL/6, and AG129 mice (*n* = 6) postinfection. (D) Viral load in liver, spleen, lung, and kidney tissues of BALB/c, C57BL/6, and AG129 mice dissected on days 3, 5, 7 (*n* = 3), and 14 (*n* = 6) postinfection. (E) The spleen tissue's pathological sections and pathological scores of BALB/c, C57BL/6, and AG129 mice dissected on Days 3, 5, 7 (*n* = 3), and 14 (*n* = 6) postinfection (the red markings indicate the site of hemorrhage, the yellow markings denote areas of inflammatory cell infiltration, and the green markings highlight the germinal centers of the spleen. Splenic hemorrhage and inflammatory cell infiltration are diffuse, with only partial locations marked in the figure).

To investigate the adaptability of the LF virus isolated from mice, BALB/c, C57BL/6, and AG129 mice were selected and subjected to the common LASV challenge method (intravenous [i.v.] tail challenge) [22], with 15 mice in each group. Each mouse was inoculated with 10^4^ plaque‐forming units (PFUs). Each group selected six mice to detect weight and body temperature at 1, 3, 5, 7, 9, 11, and 14 days postinfection and collected blood and throat swabs to monitor viremia. At 3, 5, and 7 days postinfection, three mice from each group were dissected at each time point, and all six mice from each group were dissected on Day 14 to detect viral load in tissues and observe pathological damage (Figure 4A). Long‐term observation of AG129 mouse survival rates postinfection revealed no fatalities by Day 28 postinfection, a finding that is in conformity with existing literature reports. It is speculated that the strain we isolated may have reduced toxicity due to mutations (Figure S5A). In terms of body weight, BALB/c, C57BL/6, and AG129 mice exhibited weight loss following infection, with C57BL/6 and AG129 experiencing a more pronounced decline than BALB/c (Figure 4B). Body temperature fluctuated but did not show a definite trend (Figure S5B). Viral load began to be detected 3 days postinfection, with viremia peaking in AG129 and remaining at a high level even after 14 days; in BALB/c, viremia peaked at 5 days postinfection and was undetectable in blood by Day 7, with the viral load in blood being lower than in AG129; in C57BL/6, viremia peaked at 7 days postinfection and had disappeared from the blood by Day 14, with the viral load in blood being higher than in BALB/c (Figure 4C). The viral load in throat swabs showed a similar trend to that in blood (Figure S5C). At 3, 5, 7, and 14 days postinfection, three mice were dissected at each time point, and viral load was detected in several major organs (liver, spleen, lung, and kidney) of LASV‐infected mice. In BALB/c and C57BL/6 mice, the viral load peaked at 5 days postinfection and then began to decline, reaching a lower level by Day 14. However, in AG129 mice, the viral load peaked at 5 days postinfection and remained at a high level up to Day 14, with no significant decline trend (Figure 4D). In other tissues (heart, brain, duodenum, rectum, and muscle), the viral load was higher in AG129 mice, and it could not be fully detected in BALB/c and C57BL/6 (Figure S5D). To delve deeper into the pathological effects at different intervals post‐LASV infection in BALB/c, C57BL/6, and AG129 mice, we crafted pathological tissue slices from the heart, liver, spleen, lungs, kidneys, brain, rectum, and muscle and performed pathological assessment. LASV caused varying levels of tissue harm across various organs, with the spleen enduring the most intense damage. In terms of splenic harm, the severity escalated as the infection persisted. The pathological harm in the spleen was mainly defined by bleeding, the influx of inflammatory cells, and the harm or obliteration of germinal centers (Figure 4E). In other tissues, except for the hind leg muscle, which showed no pathological damage, all exhibited some degree of pathological changes (Figures S6 and S7). However, no consistent correlation was found between the pathological scores and the duration of infection (Figure S5E).

### 2.4 Intranasal, Intramuscular, and i.p. Injection Method for Establishing a LASV‐Infected Mouse Model

**FIGURE 5:**
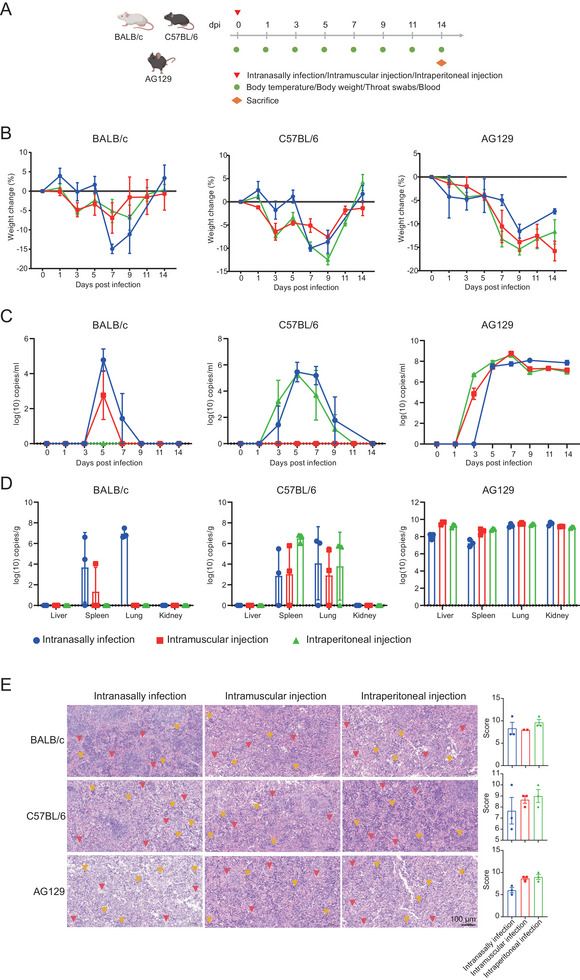
Intranasal, intramuscular (i.m.), and intraperitoneal (i.p.) infection of BALB/c, C57BL/6, and AG129 mice with LASV. (A) Schematic design of intranasal, i.m., and i.p. infection of BALB/c, C57BL/6, and AG129 mice. Body temperature, weight, blood, and throat swabs were collected every other day postinfection. Mice were dissected on day 14 postinfection. (B) Changes in body weight of BALB/c, C57BL/6, and AG129 mice (*n* = 3) postinfection. (C) Changes in viral load in the blood of BALB/c, C57BL/6, and AG129 mice (*n* = 3) postinfection. (D) Viral load in liver, spleen, lung, and kidney tissues of BALB/c, C57BL/6, and AG129 mice (*n* = 3) dissected on Day 14 postinfection. (E) The spleen tissue's pathological sections and pathological scores of BALB/c, C57BL/6, and AG129 mice (*n* = 3) dissected on Day 14 postinfection (the red markings indicate the site of hemorrhage, while the yellow markings denote areas of inflammatory cell infiltration. Splenic hemorrhage and inflammatory cell infiltration are diffuse, with only partial locations marked in the figure).

Considering that the infection levels in BALB/c and C57BL/6 mice were not as high as in AG129 mice with tail vein inoculation, to further establish a stable infection model, various inoculation methods were tested to achieve a stable LASV infection in wild‐type mice. BALB/c, C57BL/6, and AG129 mice were selected, and three inoculation methods—intranasal, intramuscular (i.m.), and i.p.—were employed for infection. Each group consisted of three mice, and each mouse was inoculated with 10^4^ PFUs. Body weight and temperature were monitored on Days 1, 3, 5, 7, 9, 11, and 14 postinfection, and blood and throat swabs were collected to monitor viral load. On Day 14 postinfection, the mice were dissected to detect viral load in tissues and observe pathological damage (Figure 5A). In C57BL/6 and AG129 mice infected with LASV via intranasal inoculation, body temperature exhibited a significant decline after 7 days, whereas no significant differences in body temperature were observed with i.m. and i.p. injection inoculations. In BALB/c mice, no significant changes in body temperature were detected (Figure S8A). Three infection methods were employed to infect BALB/c, C57BL/6, and AG129 mice. Following infection, the body weights of the mice decreased to varying extents. The intranasal infection method resulted in a more pronounced weight loss in BALB/c mice compared to the other two infection methods. In C57BL/6 and AG129 mice, the impact of the three infection methods on body weight was not significantly different (Figure 5B). The three inoculation methods had a certain impact on the viral load in the blood of BALB/c, C57BL/6, and AG129 mice. In BALB/c mice, the intranasal inoculation method resulted in the highest viremia, whereas the i.p. injection method did not cause viremia. In C57BL/6 mice, both intranasal inoculation and i.p. injection led to viremia, but i.m. injection did not (Figure 5C). From the perspective of viral load in throat swabs, the intranasal inoculation method could detect a higher level of viral load in BALB/c and C57BL/6 mice, but no significant differences were observed in AG129 mice (Figure S8B). Upon dissecting mice on Day 14 postinfection, the viral load in tissues was detected. The intranasal inoculation method was found to detect viruses in a greater number of tissues in both BALB/c and C57BL/6 mice. All three inoculation methods were capable of successfully infecting AG129 mice, with high levels of viral load detectable in various tissues throughout the body (Figure 5D, S8C). To further investigate the histopathological damage in BALB/c, C57BL/6, and AG129 mice infected through various inoculation methods, we prepared pathological tissue sections from the heart, liver, spleen, lungs, kidneys, brain, rectum, and muscle and conducted pathological scoring. LASV induced varying degrees of tissue damage in different organs, with the spleen showing the most severe damage. The pathological damage in the spleen tissue was primarily characterized by hemorrhaging, infiltration of inflammatory cells, and damage to or disappearance of germinal centers (Figure 5E). In the remaining tissues, with the exception of the hind leg muscle, which exhibited no pathological damage, varying degrees of pathological damage were observed in all other tissues (Figures S9 and S10). Different inoculation methods did not reveal significant differences in tissue damage among the various organs (Figure S8D).

### 2.5 Preliminary Exploration of LHF‐535 in LASV Infection Mouse Model

**FIGURE 6:**
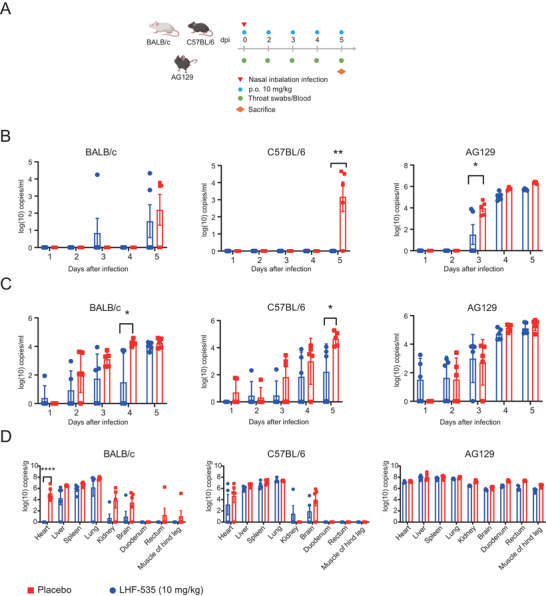
LHF‐535 drug treatment of LASV intranasal infection in BALB/c, C57BL/6, and AG129 mice. (A) Schematic design of LHF‐535 drug treatment for LASV infection in BALB/c, C57BL/6, and AG129 mice. Blood and throat swabs were collected daily postinfection. Medication was administered by gavage at a dose of 10 mg/kg daily. Mice were dissected on Day 5 postinfection. (B) Changes in viral load in the blood of BALB/c, C57BL/6, and AG129 mice (*n* = 5) after LHF‐535 drug treatment. (C) Changes in viral load in the throat swabs of BALB/c, C57BL/6, and AG129 mice (*n* = 5) after LHF‐535 drug treatment. (D) Changes in viral load after LHF‐535 drug treatment in BALB/c, C57BL/6, and AG129 mice (*n* = 5) post‐dissection. *p* < 0.05 was considered significant. The significance levels are indicated as follows: *p < 0.05; **p < 0.01; ***p < 0.001; and ****p < 0.0001.

From previous studies using mouse infection models, it was determined that the intranasal challenge method could effectively infect both BALB/c and C57BL/6 mice. To further assess the stability of this model, the drug LHF‐535, known for its ability to inhibit LASV infection [19, 20], was selected to test the established mouse model. BALB/c, C57BL/6, and AG129 mice were selected for the study. The intranasal challenge method was utilized to infect the mice, with five mice per group and each receiving a challenge of 10^4^ PFUs. Blood and throat swabs were collected to monitor viral load on Days 1, 2, 3, 4, and 5 post‐challenge. Medication was administered daily at a dose of 10 mg/kg by gavage [19, 20], starting from the day of challenge, and the mice were dissected on Day 5 post‐challenge (Figure 6A). In the drug‐treated mice, the viral load in the blood was reduced to some extent. In C57BL/6 mice, no viral load was detected in the blood of the drug treatment group at 5 dpi, showing a significant difference compared to the control group; in AG129 mice, the viral load in the blood of the drug treatment group was significantly different from the control group at 3 dpi, and the viral load in the blood of the drug treatment group was slightly lower than the control group at other time points; in BALB/c mice, the viral load in the blood of the drug treatment group was slightly lower than the control group at 5 dpi, but the difference was not significant (Figure 6B). Regarding throat swab viral load, the drug treatment group exhibited slightly lower levels compared to the control group in both BALB/c and C57BL/6 mice, with no observed difference in AG129 mice (Figure 6C). Concerning the viral load within the tissues of BALB/c and C57BL/6 mice, there were differences between the drug treatment group and the control group. Specifically, notable differences were observed in the hearts of BALB/c mice, where the drug treatment group showed a reduced viral load. In other tissues of BALB/c mice, the viral load was also slightly reduced in the drug treatment group compared to the control group. For C57BL/6 mice, the viral load in the tissues of the drug treatment group was generally lower than that of the control group, although not significantly. In contrast, no significant differences were found in AG129 mice (Figure 6D).

## 3 Discussion

Diseases caused by the LASV are characterized by a wide range of spread, strong infectivity, high mortality rate, and poor prognosis, posing a significant threat to public health [30, 31]. As China's interactions with Africa become more frequent, the risk of LF being imported into China also rises, making the study of imported cases of LASV increasingly urgent. However, China still faces a gap in the isolation of P4‐level pathogens, and the successful isolation of P4‐level pathogens is of significant importance to China. Mice are the most widely used laboratory animals due to their low cost of breeding, short generation time, and small size. Currently, there are scant reports on whether LASV infects laboratory wild‐type mice; however, with the mutation of LASV, there are limited studies on whether the newly emerging strains have become sensitive to laboratory wild‐type mice. In our study, we successfully isolated LASV and conducted an analysis of its genome and amino acid mutations, as well as the construction and evaluation of a wild‐type mouse infection model.

The successful isolation of LASV will bridge the gap in China's independent isolation of P4‐level pathogens, fulfilling the strategic needs of national biosecurity and signifying China's capacity to research and respond to the most dangerous pathogens. For the isolated pathogens, we will hold independent intellectual property rights, which can be leveraged to develop vaccines and specific medications, thereby contributing to China's strength in global public health endeavors. Comparative analysis of the sequencing data revealed that the isolated strain belongs to lineage IV, which is spreading in Guinea, Sierra Leone, Liberia, and Mali [32]. Considering that GPC is the major GP on the surface of the LASV, the entry of LASV into the host cell initially occurs through binding with the host cell receptor, followed by endocytosis into the cell interior [33]. During this process, GPC is responsible for binding to the cell receptor and the membrane fusion process. Therefore, it remains a key factor mediating viral entry across different species, and mutations on GPC can affect the process of receptor binding and membrane fusion [34]. The amino acid coding sequences of the GPC from five viruses within the same lineage were compared. The identified mutations in the LASV GPC—T59A, M96R, N114D, A133S, N219S, and S426Q—cluster in functionally critical domains, suggesting potential impacts on viral entry and immune evasion. The GP1 subunit mutations (T59A, M96R, N114D, A133S, N209S) localize to regions implicated in receptor engagement and glycan shielding. N209S has not been clearly reported, and such mutations may alter the charge, perhaps affecting interactions with host proteins. N114D and A133S disrupt predicted *N*‐linked glycosylation motifs (N‐X‐S/T), and may modify the B‐cell epitope of the isolated strain [13, 28, 35, 36]. Changes to the LASV B‐cell epitope can impact specific recognition and binding with B‐cell surface receptors or antibodies, which may attenuate glycan‐mediated antibody protection [37, 38]. This provides assistance in the research of vaccines, antibodies, and drugs [39, 40]. The M96R substitution introduces a charged residue in a hydrophobic pocket, potentially altering GP1 folding dynamics or interactions with α‐DG or LAMP1 [41]. In GP2, S426Q lies proximal to the fusion peptide; analogous mutations in arenavirus GP2 domains have been shown to modulate membrane fusion efficiency [42]. While the phenotypic consequences of these mutations require experimental validation, their spatial distribution underscores the evolutionary plasticity of LASV GPC in balancing receptor tropism and immune escape. Future studies employing pseudotyped viruses or reverse genetics could delineate their roles in viral fitness and neutralization resistance. While amino acid mutations in the L protein, NP, and Z protein are also significant for the evolution of LASV, this article primarily focuses on the exploration of GPC mutation sites and thus does not delve further into this aspect.

To further investigate whether the infectivity of the isolated strains has been enhanced after mutation, we predicted the affinity of the isolated strains' GPC for mouse, rhesus monkey, and human receptors using AlphaFold3. We discovered that the isolated strains, compared to LASV‐NC (NC‐004296.1), exhibited enhanced binding affinity of GPC to receptors from mice, rhesus monkeys, and humans. Compared to LASV‐SLE (OM791226.2), there was also an increase in the binding affinity of GPC to receptors from guinea pigs and humans. These findings suggest that the isolated strains possess greater infectivity and the ability to transmit between species than the NC and LASV strains. We speculate that this may be related to the specific mutations in isolated strains, but further experimental verification is required. In the future, results can be substantiated through techniques such as gene editing and cryo‐electron microscopy.

To further verify the adaptability of the isolated LASV in mice and establish a mouse infection model, four inoculation methods, including tail vein, intranasal, i.m., and i.p., were used to infect BALB/c, C57BL/6, and AG129 mice. Following infection, weight loss was exhibited, and viral loads were detected in throat swabs and blood. Viral loads were also maintained in major organ tissues upon dissection. It was found that after infection with the isolated LASV, the mice did not exhibit any mortality, which contradicts previous reports and suggests that the virulence of this strain may have decreased. From the perspective of pathological damage, after mice are successfully infected with LASV, the virus induces viremia and varying degrees of tissue damage in different organs, with the spleen experiencing more severe damage. The pathological damage to the spleen tissue is mainly characterized by hemorrhage, infiltration of inflammatory cells, and damage to and disappearance of germinal centers. Research Report: LASV primarily targets dendritic cells (DCs), macrophages, and endothelial cells, which are abundant in the spleen, leading to viral replication and cellular damage. After infecting DCs, LASV suppresses their maturation and antigen presentation capabilities, weakening T cell immune responses [43]. The virus replicates within macrophages, inducing the release of inflammatory factors (such as TNF‐α and IL‐6), which promote spleen damage [44]. Following LASV infection, there is an increase in spleen vascular permeability, a reduction in CD4+ T cells and B cells, and destruction of the spleen microstructure, causing excessive inflammatory responses, spleen congestion, hemorrhage, and a decrease in lymphocytes [45, 46, 47, 48]. The spleen is an important immune organ responsible for filtering pathogens and senescent blood cells from the blood; damage to the spleen may lead to a decline in immune function [49, 50]. We believe that after infecting mice, LASV may primarily cause harm by destroying their immune system. During clinical treatment, greater emphasis should be placed on the immune system's response, monitoring for any bleeding in the spleen, and preventing excessive inflammatory reactions. The liver, kidneys, and lungs are also major target organs for LASV infection. Clinical manifestations include hepatitis, acute kidney injury, and pulmonary edema. LASV efficiently replicates in hepatocytes and Kupffer cells, leading to cell apoptosis/necrosis. High levels of pro‐inflammatory factor (TNF‐α, IL‐6) expression and overactivation of NK cells and CD8+ T cells all contribute to liver damage [51, 52]. LASV can directly infect renal tubular epithelial cells, causing renal tubular necrosis; LASV can bind to glomerular endothelial cells, promoting complement activation and microthrombus formation, leading to vascular leakage and causing kidney damage [53, 54]. LASV can infect pulmonary microvascular endothelial cells, resulting in vascular leakage [55]. Pathological damage can also be observed in the heart, brain, and intestines, primarily manifesting as hemorrhaging and cellular apoptosis. In summary, LASV infection can lead to multi‐organ damage, which is the combined result of direct viral invasion and an excessive host immune response. Among all constructed mouse models, the isolated strain was capable of successfully infecting AG129 mice using all four inoculation methods. For BALB/c mice, nasal inoculation was the most consistent method. In contrast, for C57BL/6 mice, i.m. inoculation was unsuccessful, but the other three methods were effective. Consequently, BALB/c, C57BL/6, and AG129 mice could all be successfully infected with LASV through nasal inoculation. LASV demonstrates a higher and more sustained level of replication in AG129 mice, whereas in BALB/c and C57BL/6 mice, the replication level is lower and the duration is shorter.

Finally, to further validate the stability of the three LASV‐infected mouse models established via nasal inoculation, we tested the mice with LHF535. The drug exhibited a certain inhibitory effect on viral loads in blood and throat swabs and in some tissues of BALB/c and C57BL/6 mice, but the effect on AG129 mice was not significant. It is speculated that the duration of continuous administration may have been insufficient [19], and the infection level of AG129 was higher than that of C57BL/6 and BALB/c, which may not have effectively inhibited the amplification of LASV in AG129 during the early stages of administration.

Based on the aforementioned results, we have successfully isolated LASV, which belongs to lineage IV, and conducted a preliminary exploration of its molecular structure, providing evidence for the development of vaccines, antibodies, and drugs. We have also successfully constructed infection models for BALB/c, C57BL/6, and AG129 mice. The isolated strains successfully infected both wild‐type BALB/c and C57BL/6 mice, and the intranasal challenge method proved to be more effective in infecting BALB/c and C57BL/6 mice. This indicates that the strains' infectivity has increased, and they may be able to spread among mice via respiratory tract transmission. The enhanced infectivity of the virus warrants more attention and concern. Our research will bridge the gap in China's independent P4‐level pathogen isolation, meet national biosafety and strategic needs, and provide support for future studies on the evolution of the virus and the development of vaccines and drugs.

## 4 Materials and Methods

### 4.1 Animal Ethics and Biosafety Statement

BALB/c, C57BL/6, and AG129 mice were obtained from the Institute of Medical Biology, Chinese Academy of Medical Sciences, and Peking Union Medical College, Kunming, China. All animal procedures were approved by the Institutional Animal Care and Use Committee of the Institute of Medical Biology, Chinese Academy of Medical Sciences (ethics number: DWSP202410012).

### 4.2 Cell and Virus

Vero and Vero E6 cells were stored in our laboratory and cultured in Dulbecco's modified Eagle medium (DMEM) (Gibco) supplemented with 10% fetal bovine serum (FBS) (Gibco) and 1% penicillin/streptomycin at 37°C with 5% CO_2_. The viral sample originated from an imported case in Sichuan, China, in 2024, and was isolated by the Institute of Medical Biology, Chinese Academy of Medical Sciences, National Kunming High‐Level Biosafety Primate Research Center, Yunnan, China.

### 4.3 Quantification of Viral Load

RT‐qPCR was performed on the viral RNA load from throat swabs, blood, and tissues. Primers and probes based on the viral sequence were synthesized as follows [56]:

LVL forw1 AGAGCCAGCCTGATCCCAGA

LVL rev1 CACAGATAGTGGTTGTTGCACTC

LVL prb1b FAM CCGCAATTCTGCAAGAGCTGTTGGTTTGCG BHQ1

The one‐step RT‐qPCR cycling conditions were as follows: 2 min at 25°C, 15 min at 50°C, and 2 min at 95°C, followed by 40 cycles of 5 s at 95°C and 31 s at 60°C.

### 4.4 Histopathology

For histopathological evaluation, tissue samples were fixed in a 4% paraformaldehyde solution for 3–5 days. Subsequently, the tissue samples were embedded in paraffin, cut into 5‐µm sections, and stained with hematoxylin and eosin (HE). The sections were scanned using 3DHISTECH equipment. The H&E‐stained slides were scored by an experienced pathologist using the CaseViewer software. The standards for tissue scoring were based on previous studies conducted in this laboratory [57].

### 4.5 Statistical Analysis

Statistical analysis was performed using GraphPad Prism (version 9.4.0). A detailed description is provided in the figure legends. All quantitative data in this study are expressed as mean ± SEM. An unpaired two‐tailed *t*‐test was used to compare the difference between the two groups, *p* value < 0.05 was considered significant. The significance levels are indicated as follows: *p < 0.05; **p < 0.01; ***p < 0.001; and ****p < 0.0001.

## Author Contributions

Y.W, L.Z., Q.S., S.L., and X.Y. conceived this project and designed experiments; Y.Z., J.W., R.C., Y.Y., Y.F., C.T., H.Y., Q.H., W. Y., H.W., J.S., K.D., L.Y., Q.D., X.Z., H.L., M.Y., F.L., F.Z., Y.Z., and D.W. performed the experiment; S.L., Y.Y., and Y.Z. contributed significantly to data analysis and wrote the paper. All authors have read and approved the final manuscript.

## Ethics Statement

All animal procedures were approved by the Institutional Animal Care and Use Committee of the Institute of Medical Biology, Chinese Academy of Medical Sciences (ethics number: DWSP202410012).

## Conflicts of Interest

The authors declare no conflicts of interest.

## Supporting information




**Supporting Table 1**: Lassa fever case sample information.
**Supporting Fig 1**: Amino acid variation was analyzed by aligning the GPC amino acid sequence of the Isolated strain with the sequences of the 5 closest viruses and the reference genome.
**Supporting Fig 2**: Amino acid variation was analyzed by aligning the L protein amino acid sequence of the Isolated strain with the sequences of the closest viruses and the reference genome.
**Supporting Fig 3**: Amino acid variation was analyzed by aligning the Nucleoprotein and Z protein amino acid sequences of the Isolated strain with the sequences of the closest viruses and the reference genome.
**Supporting Fig 4**: Structural simulation of the binding between different LASV strains and receptors. A. Structural simulation of the binding between the NC strain GPC and α‐DG of rhesus monkeys; B. Structural simulation of the binding between the NC strain GPC and α‐DG of guinea pigs; C. Structural simulation of the binding between the SLE strain GPC and α‐DG of rhesus monkeys; D. Structural simulation of the binding between the SLE strain GPC and α‐DG of guinea pigs; E. Structural simulation of the binding between the isolated strain GPC and α‐DG of rhesus monkeys; F. Structural simulation of the binding between the isolated strain GPC and α‐DG of guinea pigs. Deep blue represents GPC, sky blue represents α‐DG, and red represents interaction sites. Iptm represents interaction score (the higher the score, the stronger the interaction), ptm represents structural prediction accuracy (the higher the accuracy, the more accurate), and the sum of the two indicates the strength of the final receptor‐ligand binding ability.
**Supporting Fig 5**: i.v. tail infection method infecting BALB/c, C57BL/6, and AG129 mice with LASV. Survival rate of AG129 mice(n = 6) 28 days after i.v. tail infection; B. Changes in body temperature of BALB/c, C57BL/6, and AG129 mice after infection; C. Changes in viral load in the throat swabs of BALB/c, C57BL/6, and AG129 mice after infection; D. Viral load in heart, brain, duodenum, and rectum of BALB/c, C57BL/6, and AG129 mice, dissected on days 3, 5, 7, and 14 after infection. E. The lung, liver, muscle of hind leg, brain, kidney, heart, and rectum tissues’s pathological scores of BALB/c, C57BL/6, and AG129 mice, dissected on days 3, 5, 7, and 14 after infection.
**Supporting Fig 6**: The heart, liver, and lung tissues’s pathological sections of BALB/c, C57BL/6, and AG129 mice, dissected on days 3, 5, 7, and 14 after infection.
**Supporting Fig 7**: The kidney, brain, and rectum tissues’s pathological sections of BALB/c, C57BL/6, and AG129 mice, dissected on days 3, 5, 7, and 14 after infection.
**Supporting Fig 8**: Intranasally, i.m., and i.p. infecting BALB/c, C57BL/6, and AG129 mice with LASV. Changes in body temperature of BALB/c, C57BL/6, and AG129 mice after infection; B. Changes in viral load in the throat swabs of BALB/c, C57BL/6, and AG129 mice after infection; C. Viral load in heart, brain, duodenum, and rectum of BALB/c, C57BL/6, and AG129 mice, dissected on day 14 after infection. D. The lung, liver, muscle of hind leg, brain, kidney, heart, and rectum tissues’s pathological scores of BALB/c, C57BL/6, and AG129 mice, dissected on day 14 after infection.
**Supporting Fig 9**: The heart, liver, and lung tissues's pathological sections of BALB/c, C57BL/6, and AG129 mice dissected on day 14 postinfection.
**Supporting Fig 10**: The kidney, brain, and rectum tissues’s pathological sections of BALB/c, C57BL/6, and AG129 mice were dissected on day 14 postinfection.

## Data Availability

All study data are included in the article and supplementary information. The complete Isolated‐L and Isolated‐S sequences have been uploaded to the Genbase database with accession numbers Isolated‐L (C_AA084677.1) and Isolated‐S (C_AA084676).
