# Supplementary material for: The Biological Characteristics and Mouse Model of Lassa Virus From the First Imported Case in China
Source: MedComm (2020). 2025 Aug 3;6(8):e70315. doi: 10.1002/mco2.70315 (PMC12318830; doi:10.1002/mco2.70315)
Supplement: Supplementary file 1 — Supporting Table 1: Lassa fever case sample information. Supporting Fig 1: Amino acid variation was analyzed by aligning the GPC amino acid sequence of the Isolated strain with the sequences of the 5 closest viruses and the reference genome. Supporting Fig 2: Amino acid variation was analyzed by aligning the L protein amino acid sequence of the Isolated strain with the sequences of the closest viruses and the reference genome. Supporting Fig 3: Amino acid variation was analyzed by aligning the Nucleoprotein and Z protein amino acid sequences of the Isolated strain with the sequences of the closest viruses and the reference genome. Supporting Fig 4: Structural simulation of the binding between different LASV strains and receptors. A. Structural simulation of the binding between the NC strain GPC and α‐DG of rhesus monkeys; B. Structural simulation of the binding between the NC strain GPC and α‐DG of guinea pigs; C. Structural simulation of the binding between the SLE strain GPC and α‐DG of rhesus monkeys; D. Structural simulation of the binding between the SLE strain GPC and α‐DG of guinea pigs; E. Structural simulation of the binding between the isolated strain GPC and α‐DG of rhesus monkeys; F. Structural simulation of the binding between the isolated strain GPC and α‐DG of guinea pigs. Deep blue represents GPC, sky blue represents α‐DG, and red represents interaction sites. Iptm represents interaction score (the higher the score, the stronger the interaction), ptm represents structural prediction accuracy (the higher the accuracy, the more accurate), and the sum of the two indicates the strength of the final receptor‐ligand binding ability. Supporting Fig 5: i.v. tail infection method infecting BALB/c, C57BL/6, and AG129 mice with LASV. Survival rate of AG129 mice(n = 6) 28 days after i.v. tail infection; B. Changes in body temperature of BALB/c, C57BL/6, and AG129 mice after infection; C. Changes in viral load in the throat swabs of BALB/c, C57BL/6, and AG129 mic [file MCO2-6-e70315-s001.docx]

**The biological characteristics and mouse model of Lassa virus from first imported case in China**

Yanan Zhou^1#^, Junbin Wang^1#^, Ranran Cao^2#^, Yun Yang^1#^, Yuliang Feng^2#^, Cong Tang^1^, Hao Yang^1^, Qing Huang^1^, Wenhai Yu^1^, Haixuan Wang^1^, Jiandong Shi^1^, Kaiyun Ding^1^, Longhai Yuan^1^, Qing Dai^1^, Xingping Zhao^1^, Haiyan Li^1^, Mengli Yang^1^, Fangyu Luo^1^, Fanli Zhu^1^, Yong Zhang^1^, Daoju Wu^1^, Xiaorong Yang^2^, Shuaiyao Lu^1,3,4*^, Qiangming Sun^1,3,4*^, Li Zhang^2*^, Youchun Wang^1,3,4*^

1 National Kunming High‐Level Biosafety Primate Research Center，State Key Laboratory of Respiratory Health and Multimorbidity, Institute of Medical Biology, Chinese Academy of Medical Sciences and Peking Union Medical College, Kunming, China; 2 Sichuan Center for Disease Control and Prevention, Chengdu, China; 3 Key Laboratory of Pathogen Infection Prevention and Control (Peking Union Medical College), Ministry of Education, Beijing, China; 4 Yunnan Key Laboratory of Cross-Border Infectious Disease Control and Prevention and Novel Drug Development, Kunming, China.

* Correspondence: Shuaiyao Lu (lushuaiyao-km@163.com) | Qiangming Sun (qsun@imbcams.com.cn) | Li Zhang (657096242@qq.com) | Youchun Wang [(wangyc@nifdc.org.cn)](mailto:(wangyc@nifdc.org.cn))

# These authors contribute equally to this study.

**This file includes:**

**Table S1**

**Figure S1-S10**

**Materials and Methods**

**Table S1 Lassa fever case sample information**

| Sample number | Sample type | Sampling time | Ct value | Inactivate or not | Amount | Sending unit | Receiving unit | Sample receiving time |
| --- | --- | --- | --- | --- | --- | --- | --- | --- |
| LSR24-07 | Whole blood | 2024.08.01 | 31.45 | No | 1 | Sichuan Center for Disease Control and Prevention | Chinese Academy of Medical Sciences & Peking Union Medical College Institute of Medical Biology | 2024.10.14 |
| LSR24-27-3 | Urine | 2024.08.12 | 31.62 | No | 1 | Sichuan Center for Disease Control and Prevention | Chinese Academy of Medical Sciences & Peking Union Medical College Institute of Medical Biology | 2024.10.14 |
| LSR-24-64-3 | Urine | 2024.08.21 | 33.7 | No | 1 | Sichuan Center for Disease Control and Prevention | Chinese Academy of Medical Sciences & Peking Union Medical College Institute of Medical Biology | 2024.10.14 |
| LSR-24-125 | Urine | 2024.09.10 | 36.57 | No | 1 | Sichuan Center for Disease Control and Prevention | Chinese Academy of Medical Sciences & Peking Union Medical College Institute of Medical Biology | 2024.10.14 |


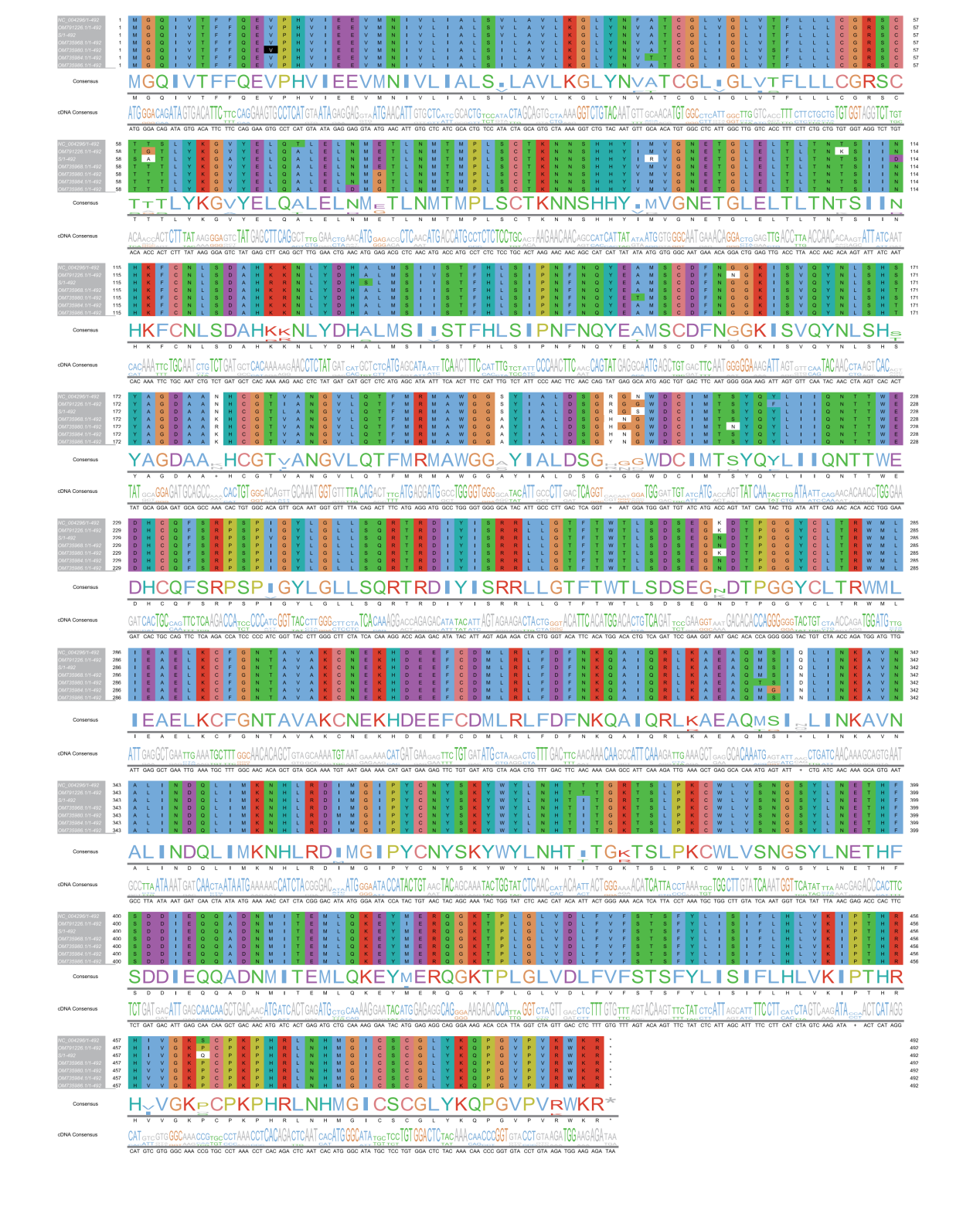


**Figure S1. Amino acid variation was analyzed by aligning the GPC amino acid sequence of Isolated strain with the sequences of the 5 closest viruses and the reference genome.**

**
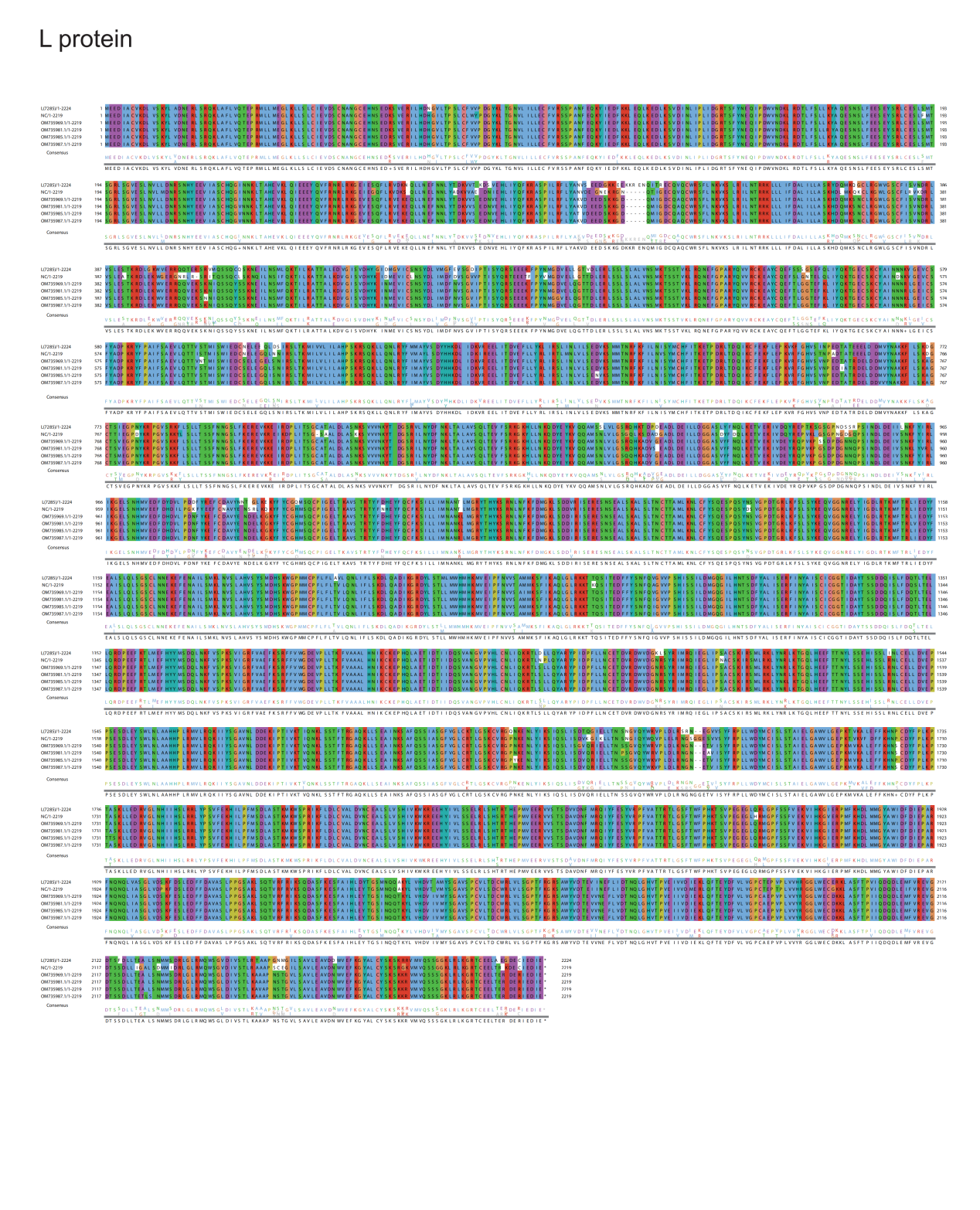
**

**Figure S2. Amino acid variation was analyzed by aligning the L protein amino acid sequence of Isolated strain with the sequences of the closest viruses and the reference genome.**

**
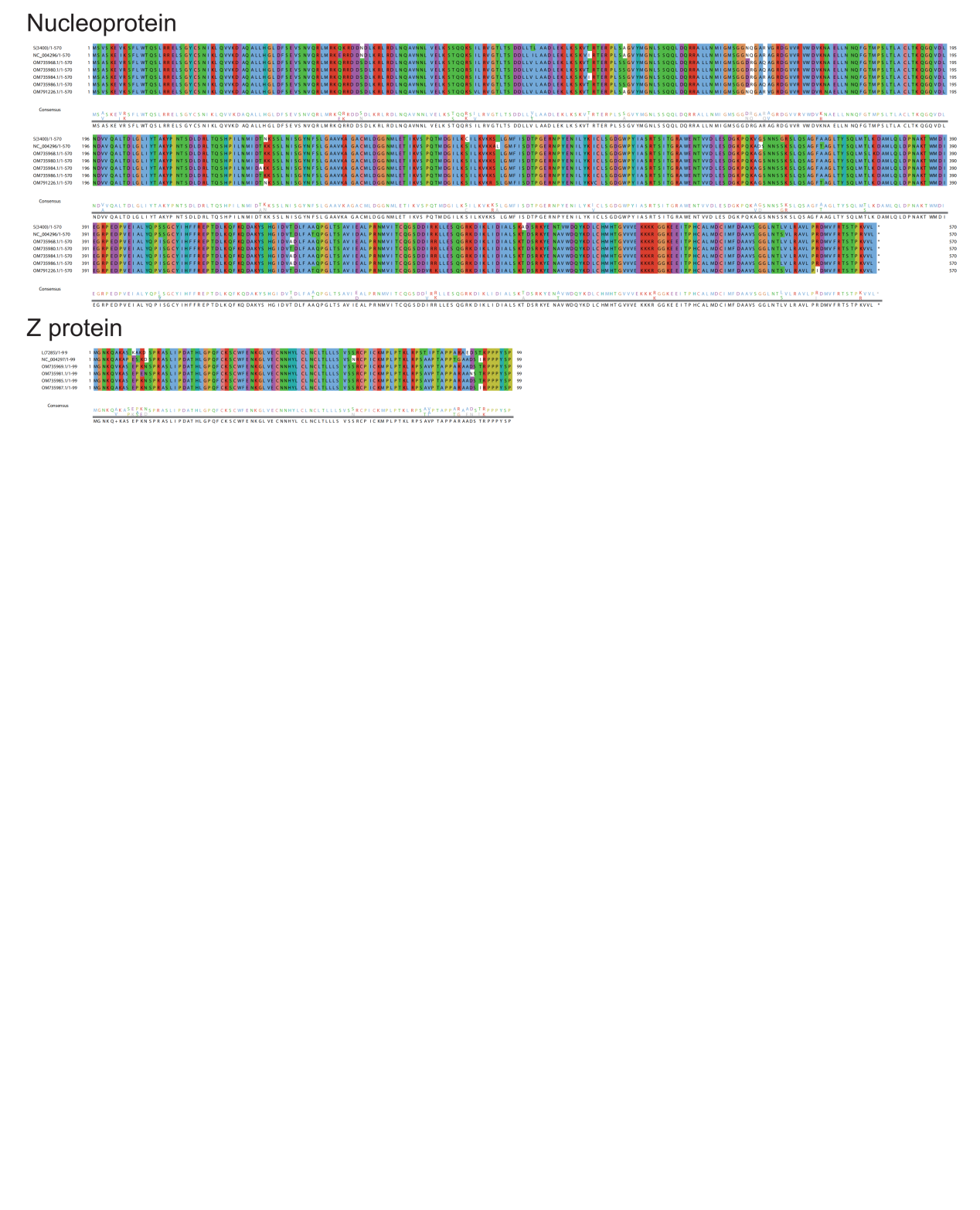
**

**Figure S3. Amino acid variation was analyzed by aligning the Nucleoprotein and Z protein amino acid sequence of Isolated strain with the sequences of the closest viruses and the reference genome.**


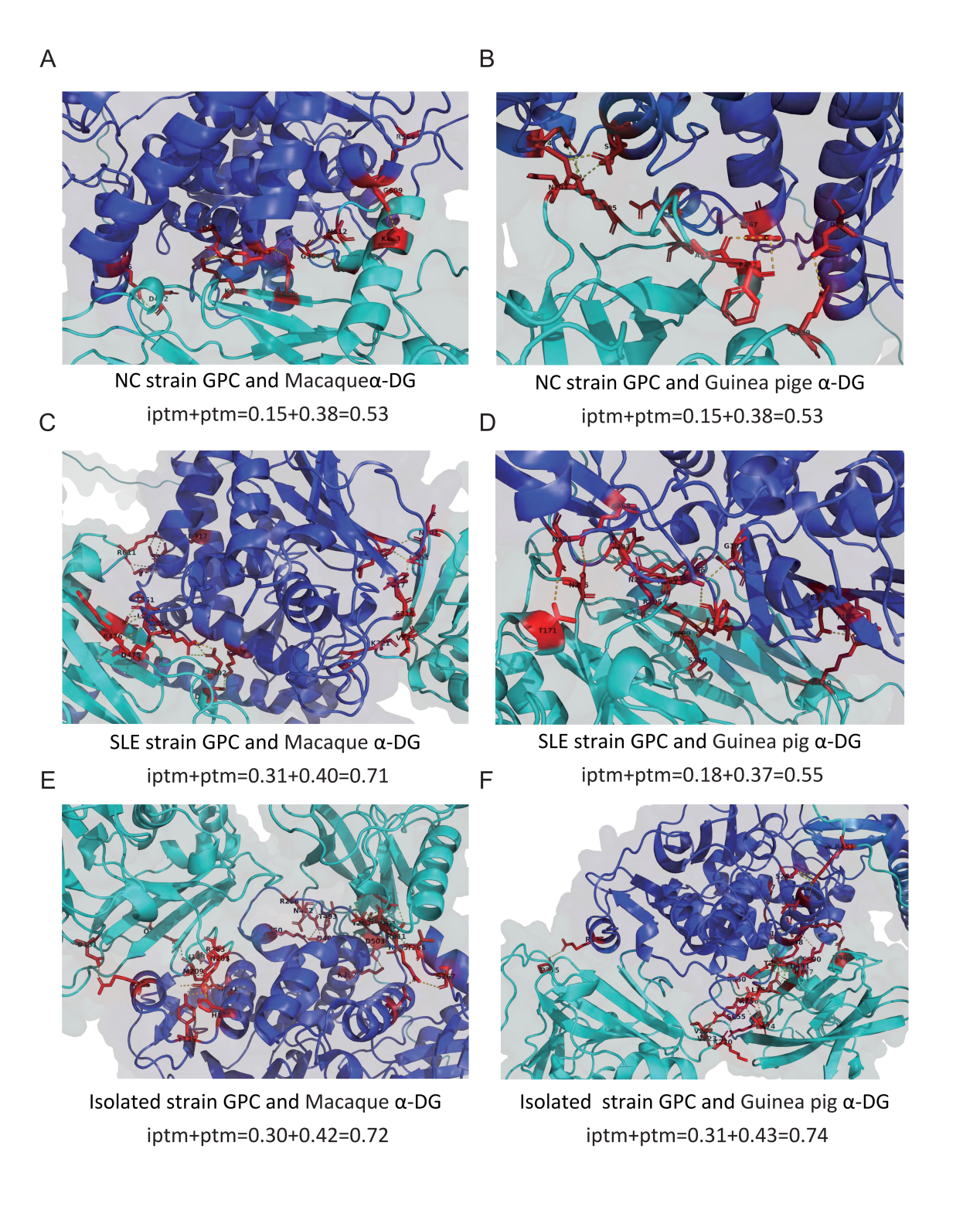


**Figure S4. Structural simulation of the binding between different LASV strains and receptors.**

A. Structural simulation of the binding between the NC strain GPC and α-DG of rhesus monkeys; B. Structural simulation of the binding between the NC strain GPC and α-DG of guinea pigs; C. Structural simulation of the binding between the SLE strain GPC and α-DG of rhesus monkeys; D. Structural simulation of the binding between the SLE strain GPC and α-DG of guinea pigs; E. Structural simulation of the binding between the isolated strain GPC and α-DG of rhesus monkeys; F. Structural simulation of the binding between the isolated strain GPC and α-DG of guinea pigs.

Deep blue represents GPC, sky blue represents α-DG, and red represents interaction sites. Iptm represents interaction score (the higher the score, the stronger the interaction), ptm represents structural prediction accuracy (the higher the accuracy, the more accurate), and the sum of the two indicates the strength of the final receptor-ligand binding ability.


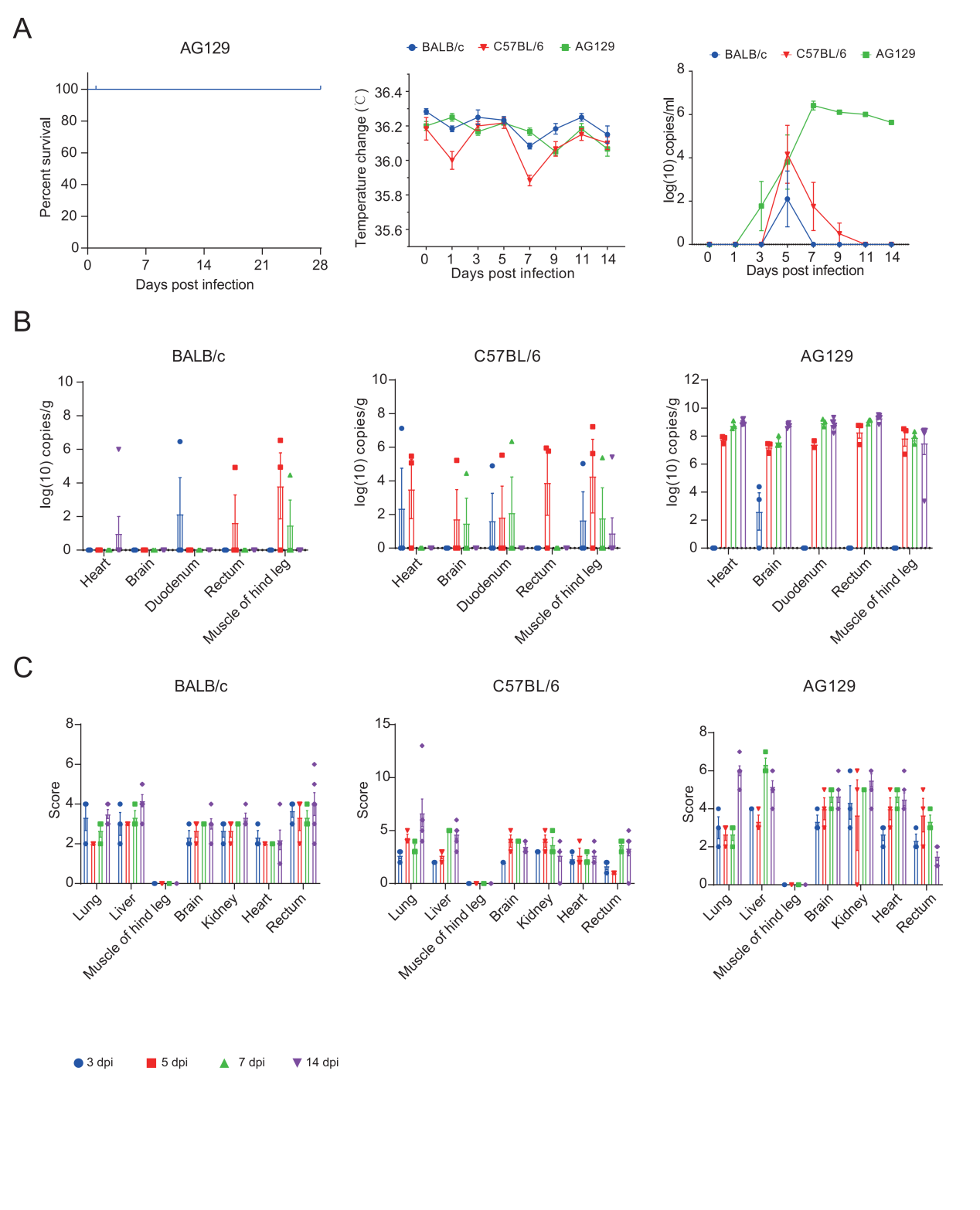


**Figure S5. Intravenous tail infection method infecting BALB/c, C57BL/6, and AG129 mice with LASV.**

1. Survival rate of AG129 mice(n=6) 28 days after intravenous tail infection; B. Changes in body temperature of BALB/c, C57BL/6, and AG129 mice after infection; C. Changes in viral load in the throat swabs of BALB/c, C57BL/6, and AG129 mice after infection; D. Viral load in heart, brain, duodenum, and rectum of BALB/c, C57BL/6, and AG129 mice, dissected on days 3, 5, 7, and 14 after infection. E. The lung,liver,muscle of hind leg,brain,kidney,heart and rectum tissues’s pathological scores of BALB/c, C57BL/6, and AG129 mice, dissected on days 3, 5, 7, and 14 after infection.


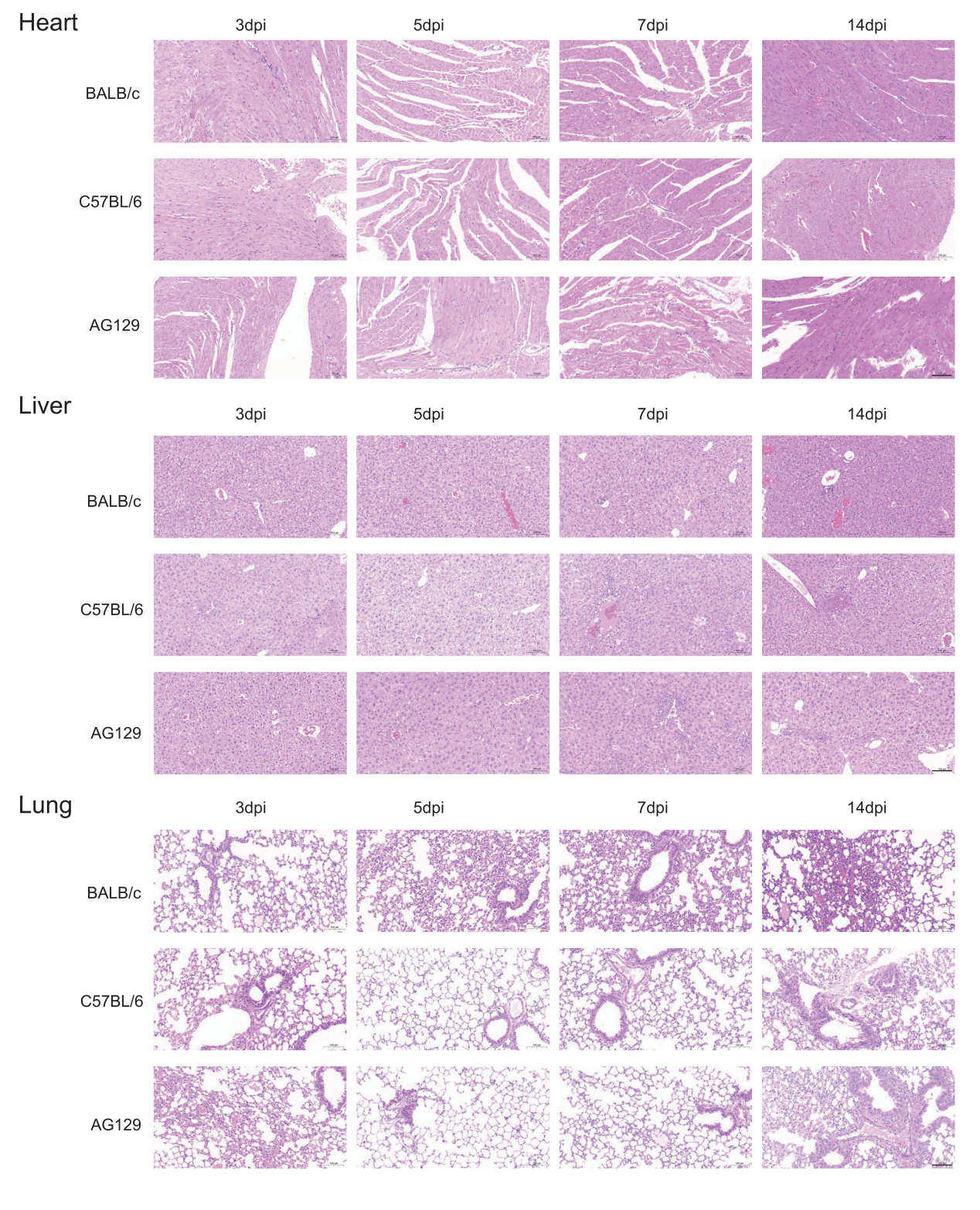


**Figure S6. The heart, liver and lung tissues’s pathological sections of BALB/c, C57BL/6, and AG129 mice, dissected on days 3, 5, 7, and 14 after infection.**

**
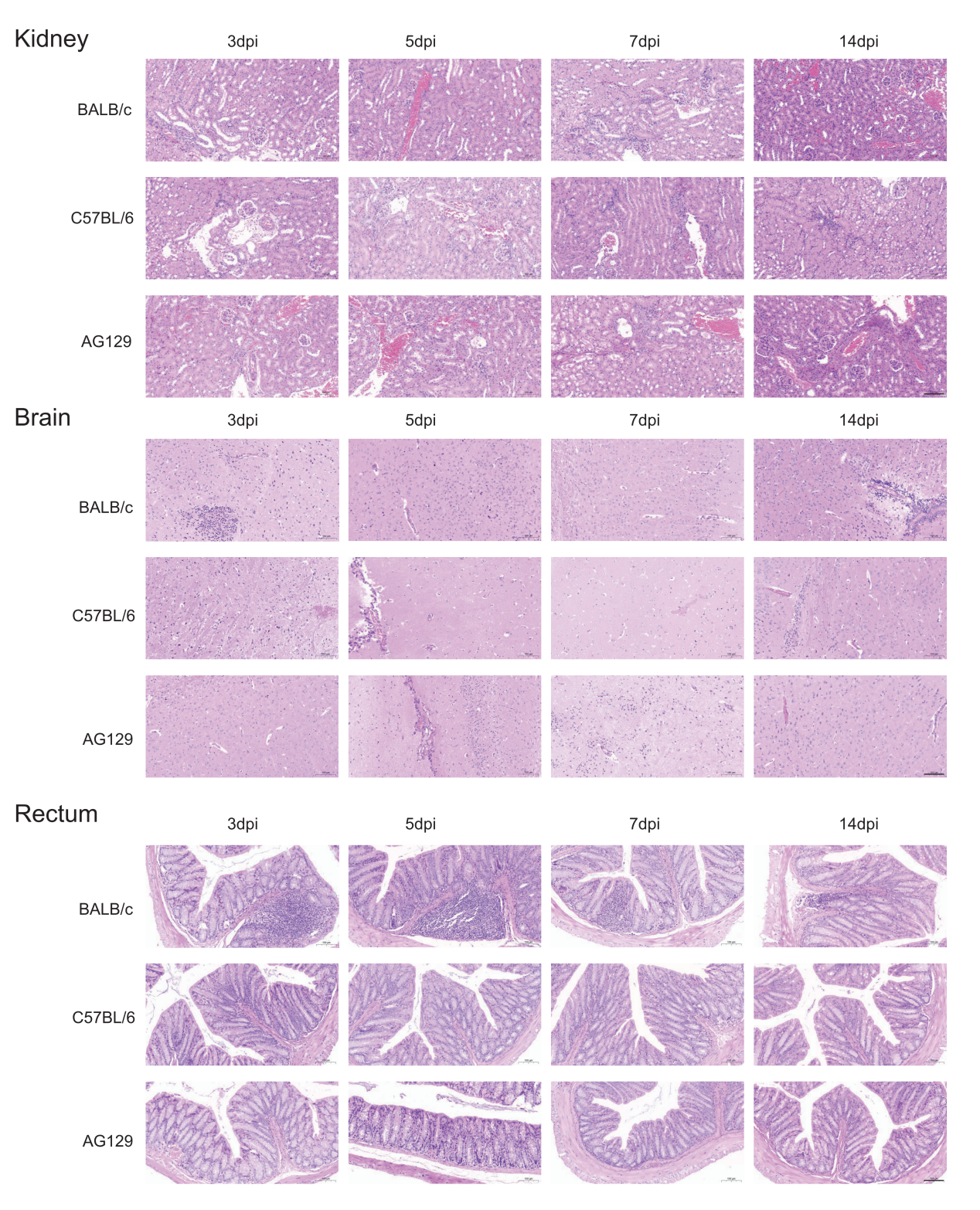
**

**Figure S7. The kidney, brain and rectum tissues’s pathological sections of BALB/c, C57BL/6, and AG129 mice, dissected on days 3, 5, 7, and 14 after infection.**


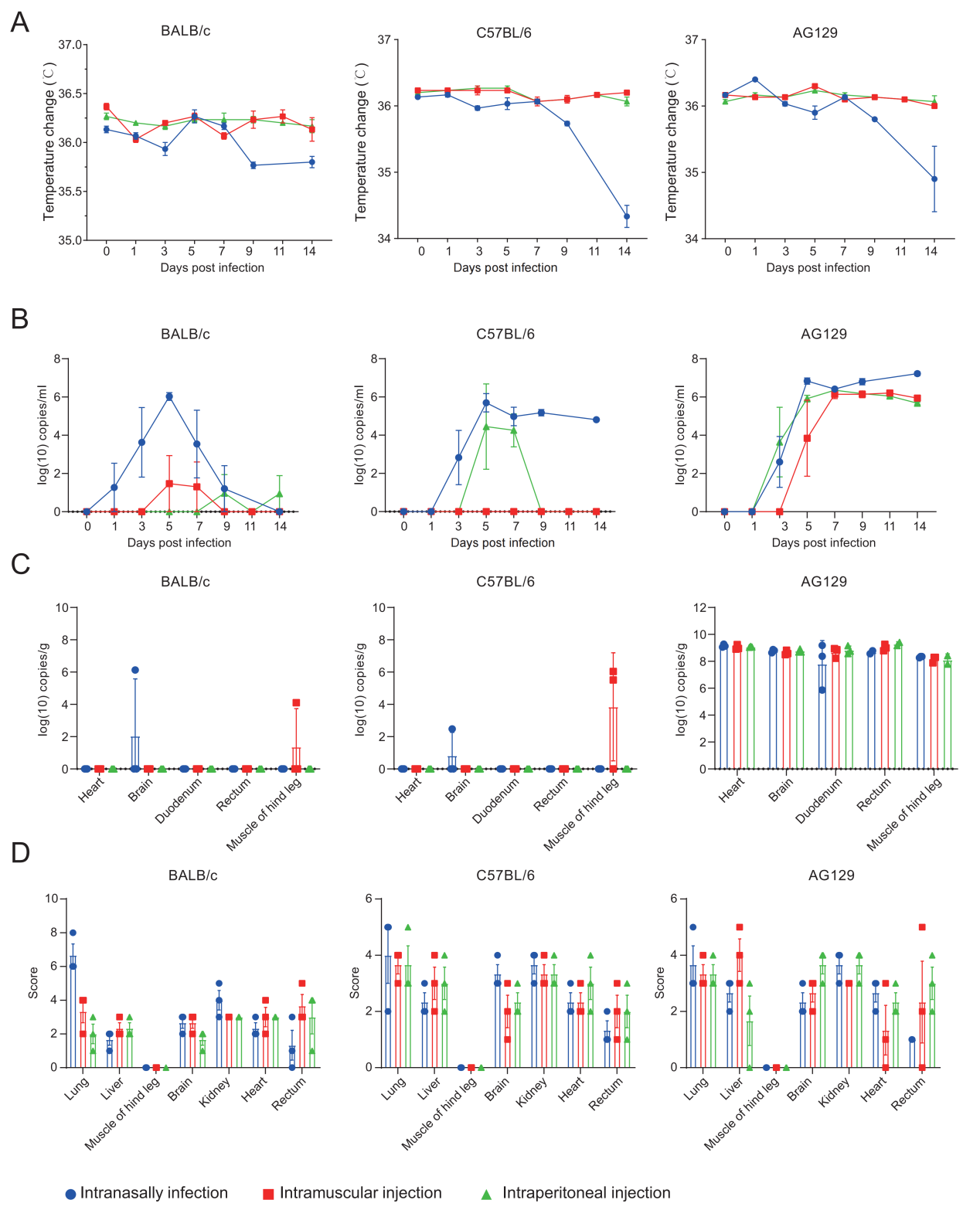


**Figure S8. Intranasally, i.m., and i.p. infecting BALB/c, C57BL/6, and AG129 mice with LASV.**

1. Changes in body temperature of BALB/c, C57BL/6, and AG129 mice after infection; B. Changes in viral load in the throat swabs of BALB/c, C57BL/6, and AG129 mice after infection; C. Viral load in heart, brain, duodenum, and rectum of BALB/c, C57BL/6, and AG129 mice, dissected on day 14 after infection. D. The lung,liver,muscle of hind leg,brain,kidney,heart and rectum tissues’s pathological scores of BALB/c, C57BL/6, and AG129 mice, dissected on day 14 after infection.


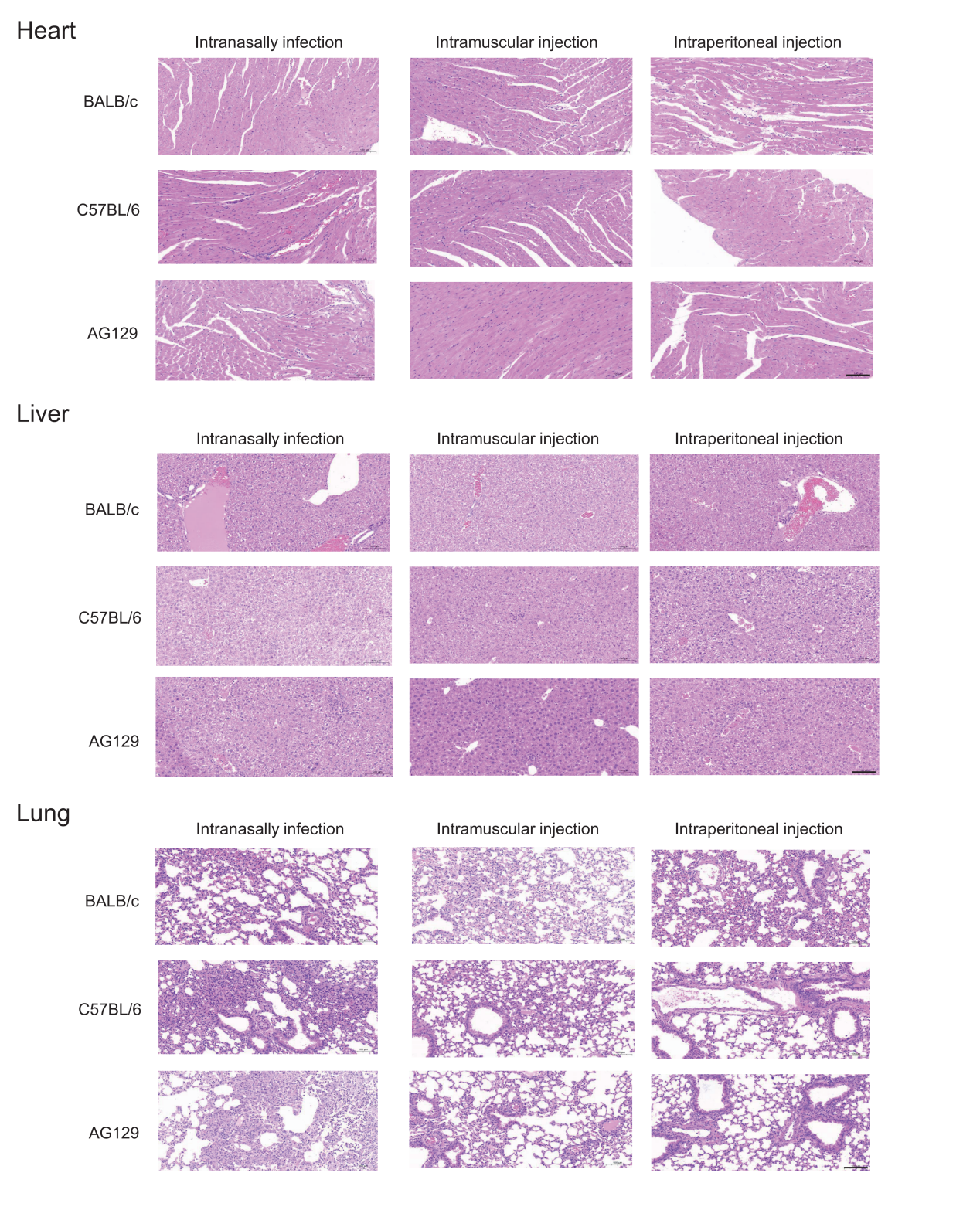


**Figure S9. The heart, liver and lung tissues’s pathological sections of BALB/c, C57BL/6, and AG129 mice dissected on day 14 post-infection.**


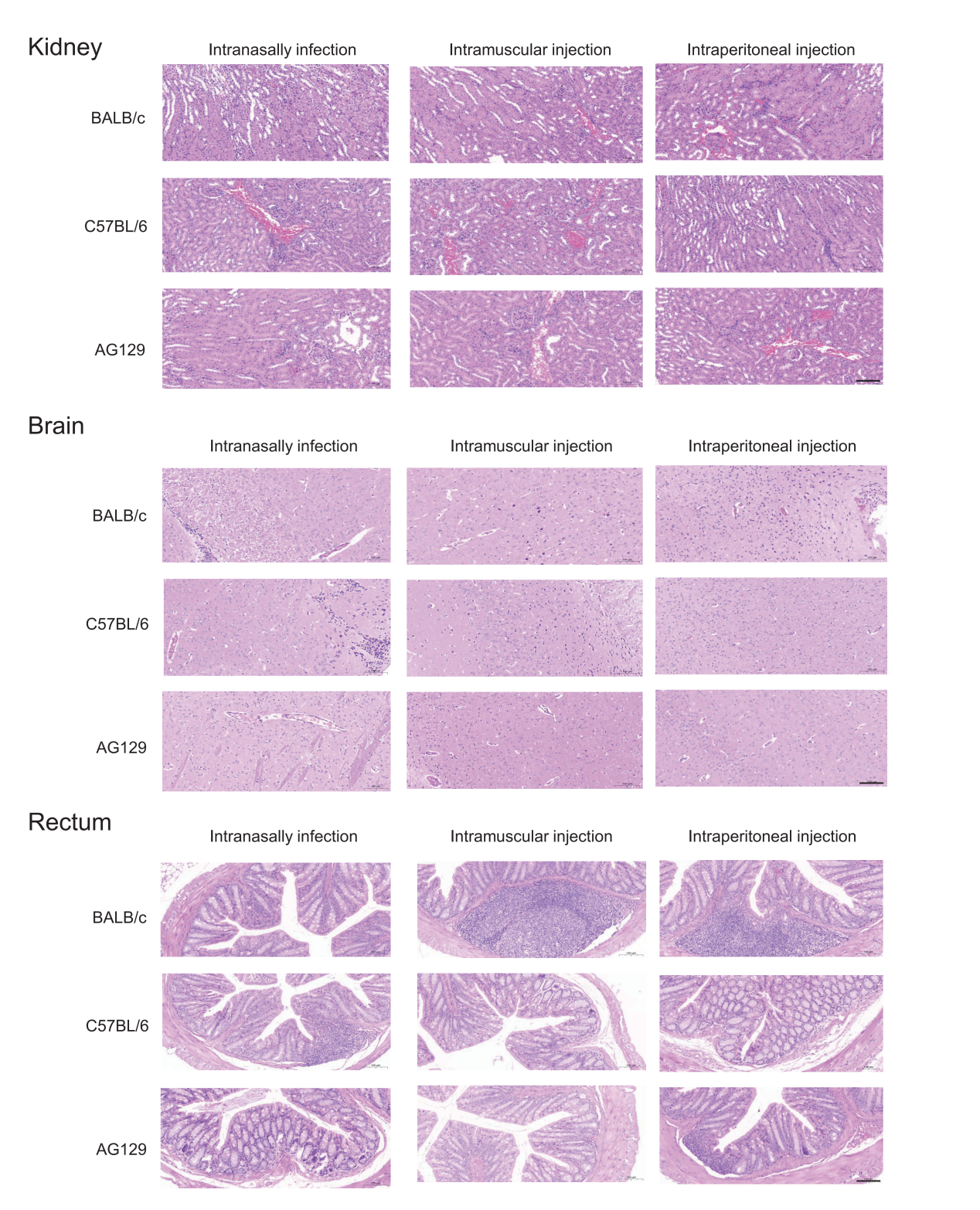


**Figure S10. The kidney, brain and rectum tissues’s pathological sections of BALB/c, C57BL/6, and AG129 mice dissected on day 14 post-infection.**

**Materials and methods**

**Animal husbandry**

All animals were kept in a specific pathogen-free (SPF) environment and conducted in accordance with the Guidelines for the Care and Use of Laboratory Animals by the National Institutes of Health. All experiments involving the infection of LASV were carried out in a biosafety level 4 (BSL4) facility at the Institute of Medical Biology, Chinese Academy of Medical Sciences, National Kunming High-level Biosafety Primate Research Center, Yunnan, China.

**Isolation and identification of LASV**

The viral strain was isolated through intracerebral injection in 3-day-old AG129 suckling mice for serial passage. Five days post-inoculation, we harvested the brains of the suckling mice, homogenized them in PBS, centrifuged the mixture, and subsequently utilized the supernatant for further passaging and RT-qPCR to detect viral load. After two rounds of subculturing, LASV-positive samples were identified in the brains of neonatal mice, and the virus was subsequently passaged in Vero and Vero E6 cells. The virus was propagated on Vero cells, identified via reverse transcription polymerase chain reaction (RT–PCR), transmission electron microscopy, and sequencing, and titrated via a plaque assay. The transmission electron microscopy method involves inactivating and fixing the LASV solution with 4% paraformaldehyde (with a formaldehyde to virus solution ratio of 1:1). After three days in a 4°C refrigerator, the virus is concentrated and purified using a 100kd ultrafiltration tube to ensure a concentration of virus particles greater than 10^6 particles/ml. The sample is then processed with negative staining and observed under an electron microscope. For the titer determination method, Vero cells are seeded one day in advance to allow them to grow to a monolayer in a 6-well plate (500,000 cells per well). Prior to the experiment, the cells are washed twice with PBS to remove serum residues. The virus is serially diluted 10-fold with DMEM medium and inoculated into the 6-well plate, with 500μl per well. The plate is then incubated in a 37°C incubator for 1-2 hours for adsorption. After adsorption, the virus solution is aspirated, the wells are washed once with PBS, and 2ml of methylcellulose semi-solid culture medium is added to each well. The plate is cultured in a 37°C, 5% CO2 incubator for 7 days. Following this period, the semi-solid culture medium is aspirated, the wells are fixed with 4% paraformaldehyde for 30 minutes, and the paraformaldehyde is then aspirated. The wells are stained with 0.1% crystal violet for 10 minutes to count and calculate the virus titer.

**Virus Genome Sequencing and Analysis**

The isolated virus was cultured in Vero cells for 5 days, after which the cell supernatant was mixed with 3 volumes of Trizol Reagent (15596062) to inactivate the virus. Ribonucleic acid(RNA) was extracted from the virus samples and sequenced using next-generation sequencing technology (DNBSEQ-T7). The raw data underwent quality control using fastp (version 0.23.2), with the specific quality control steps as follows: 1. Removal of linker sequences from reads and filtering out reads with insertions shorter than 15 bp. 2. Filtering out low-quality reads, and obtaining clean reads (98.16%) by filtering out reads containing more than five N bases. After quality control, the data were aligned to the host genome (Mmul 10), resulting in unmapped reads. These unmapped reads were assembled without references using Trinity (v2.15.2), and the assembly results were then blasted against the supplied reference genome sequence (L: NC 004297.1, S: NC 004296.1) with parameters (-evalue 1e-5, -max 3, -max 5) to extract the matching sequences. The complete Isolated-L and Isolated-S sequences have been uploaded to the Genbase database with the accession numbers (C_AA084677.1) and (C_AA084676). Sequence alignment and phylogenetic tree construction were performed using MEGA 11, with the sequence alignment algorithm using Clustal W and the phylogenetic tree building model employing General Time Reversible (GTR), Rates among Sites using Gamma distribution with Invariant Sites, and the Test of Phylogeny using Bootstrap. Initial sequence selection was conducted through BLASTn analysis against the NCBI nucleotide database, identifying top 30 closely matching sequences from clinically isolated strains (top hits by E-value and percent identity) along with relevant reference sequences. These sequences were aligned using MUSCLE in MEGA 11 (v11.0.13) with default parameters, followed by manual refinement of ambiguous regions. The optimal substitution model was determined through maximum likelihood estimation, with the GTR+G+I model selected for nucleotide sequences based on Bayesian Information Criterion scores. Phylogenetic reconstruction was performed using maximum likelihood methods with 1000 bootstrap replicates, employing Neighbor-Joining initial trees and Subtree-Pruning-Regrafting optimization. The multiple sequence alignment results were imported into Jalview (v2.11.3) for enhanced visualization and annotation. Amino acid variation was analyzed by aligning the amino acid sequence of the isolated strain with the sequences of the closest viruses(2019, Kamasseh-08476 (OM791226.1), Bapodia-00204 (OM735980.1), Bapodia-00187 (OM735984.1), Bapodia-00303 (OM735968.1), and Bapodia-00106(OM735986.1)) and the reference genome. The 492 amino acid coding sequence of the isolated strain's GPC protein was aligned with the sequences of the 5 closest viruses and the reference genome. The L protein, Nucleoprotein, and Z protein of the isolated strain, which respectively use 2224, 570, and 99 amino acid coding sequences, were aligned with the sequences of the closest viruses and the reference genome.

**Receptor Affinity Analysis**

The LASV GPC sequences from various strains utilized in this receptor binding analysis were sourced from NCBI, including LASV-NC (NC_004296.1), LASV-SLE (OM791226.1), and LASV-Isolate sequences obtained through sequencing. The amino acid sequences of LASV receptor α-dystroglycan(α-DG) were sourced from the Uniport database, including those from mouse (Q62165), human (Q14118), macaque (F6RU27), and guinea pig (A0A286XNA0). On the Alphafold3 online platform (alphafoldserver.com), two sequence input boxes were added, into which the GPC sequences of different strains and the α-DG sequences of different species were entered respectively. The structure prediction and the interaction between the two were then initiated, with the results of the highest confidence level being used as the model for further analysis. After downloading the predicted results, the PyMol software (v4.60) was used for visual analysis. Specifically, the structures of GPC and α-DG were represented in different colors (deep blue for GPC, sky blue for α-DG), and potential interaction sites between the two structures were identified and marked in red. Finally, the overall structural diagram and the interaction diagram were rendered and exported. Interface predicted template modeling(iptm) represents the interaction score (the higher the score, the stronger the interaction), and predicted template modeling(ptm) represents the accuracy of structural prediction (the higher the accuracy, the more precise). The sum of the two indicates the strength of the final receptor-ligand binding capability.

**Animal experimental**

Select BALB/c, C57BL/6, and AG129 mice and use the common Lassa virus challenge method (intravenous tail challenge) to infect them, with 15 mice per group, and infect each mouse with 10^4 plaque-forming units (PFUs). Each group selected 6 mice to detect weight and body temperature at 1, 3, 5, 7, 9, 11, and 14 days post-infection, and collected blood and throat swabs to monitor viremia. On days 3, 5, and 7 post-challenge, dissect 3 mice from each time point, and on day 14 dissect all 6 mice from each group to detect viral load in tissues and observe pathological damage. For the survival rate observation experiment, six AG129 mice were selected and infected via tail vein injection with 10^4 PFUs of the virus. The survival status of the mice was monitored until 28 days post-challenge. Select BALB/c, C57BL/6, and AG129 mice and use three challenge methods: intranasal, intramuscular, and intraperitoneal to challenge with LASV, with 3 mice per group, and infect each mouse with 10^4 PFUs. Monitor weight and body temperature on days 1, 3, 5, 7, 9, 11, and 14 post-challenge, and collect blood and throat swabs to monitor viral load. On day 14 post-challenge, dissect to detect viral load in tissues and observe pathological damage. Isoflurane gas anesthesia was used to anesthetize the mice. Following anesthesia, the mice's body weight was measured using a precision electronic balance with a 0.01g accuracy and recorded. An infrared thermometer was employed to detect the body temperature of the mice. Subsequently, sterile cotton swabs or swabs suitable for small animals were used to gently insert into the pharynx of the mouse, rotate the swab lightly to scrape, and then slowly remove it and place it into a centrifuge tube containing 800μl of Trizol. After anesthesia, blood was collected from the retro-orbital venous plexus of the mice. The capillary was held and inserted obliquely about 5mm from the inner corner of the eye, gently rotated and then withdrawn. The blood flowed into the tube by capillary action. 10μl of blood was taken and added to three times the volume of Trizol. Following blood collection, dry cotton balls were used to compress the eyeball for hemostasis. After deep anesthesia, the mice were exposed to the virus. The challenge volume was 100μl per mouse. The virus solution was prepared with PBS, ensuring that each 100μl solution contained 10^4 PFUs of the virus. When challenging the virus through intranasal inoculation, one hand was used to stabilize the mouse, while the other hand utilized a pipette to administer 100μl of virus solution, observing the mouse's breathing rhythm, and slowly dripping the virus into the mouse's nostrils.When challenging through the tail vein, the mouse was fixed on the mouse tail vein injection instrument, and then 100μl of virus was injected into the mouse's tail vein with a syringe. When challenging through intraperitoneal and intramuscular injection, one person held the mouse, and the other person used a syringe to administer 100μl of virus and injected it into the mouse's peritoneal cavity and the lateral muscle of the mouse's thigh.

**Drug evaluation**

LHF-535 was suspended in 0.5% Methocel E15 and 1% Tween 80^[19,20]^. BALB/c, C57BL/6, and AG129 mice were selected, and the intranasal inoculation method was used for infection, with 5 mice per group, and each mouse was inoculated with 10^4 plaque-forming units (PFUs). Blood and throat swabs were collected to monitor viral load on days 1, 2, 3, 4, and 5 post-inoculation. Post-inoculation, medication was administered daily at a dose of 10mg/kg by gavage, and on day 5 post-inoculation, tissues were dissected to detect viral load.

**Nucleic acid extraction and RT-qPCR**

Blood samples were mixed with Trizol at a ratio of 1:3. Tissue samples were weighed, added to 800μl of Trizol, and homogenized for inactivation. Throat swabs were inactivated with 800μl of Trizol. RNA was extracted using the UPure Virus RNA Plus Kit (Cat# M2006P-A96, Biokeystone, China), and the extracted nucleic acids were used for RT-qPCR detection. RT-qPCR detection was conducted using the TaqMan Fast Virus 1-Step Master Mix (Cat# 4444432, Thermo Fisher Scientific, USA) on a CFX384 Touch Real-Time PCR Detection System (Bio-Rad, USA). Preparation of the standard substance: Utilizing the known coding sequence of the LASV Z protein, RNA fragments are synthesized to serve as the standard. Subsequently, the NEBioCalculator is employed to determine the copy number of the standard. Ten-fold serial dilutions of the standard are performed, spanning 7-8 dilution levels. Both the standard and samples are loaded onto the machine simultaneously, employing the same primers and probes. After the RT-qPCR reaction was completed, the number of viral copies was calculated based on the standards. The detection limit is defined as when the Relative Fluorescence Units (RFU) are less than 250 and the CT value exceeds 40.
